# High molecular weight DNA extraction methods lead to high quality filamentous ascomycete fungal genome assemblies using Oxford Nanopore sequencing

**DOI:** 10.1099/mgen.0.000816

**Published:** 2022-04-19

**Authors:** Celine Petersen, Trine Sørensen, Klaus R. Westphal, Lavinia I. Fechete, Teis E. Sondergaard, Jens L. Sørensen, Kåre L. Nielsen

**Affiliations:** ^1^​ Department of Chemistry and Bioscience, Fredrik Bajers Vej 7H, 9220 Aalborg, Denmark, Aalborg University; ^2^​ Department of Chemistry and Bioscience, Niels-Bohrs Vej 8, 6700 Esbjerg, Denmark, Aalborg University

**Keywords:** Long-read sequencing, MinION, DNA extraction, high molecular weight DNA, genome assembly, filamentous fungi, ascomycete

## Abstract

During the last two decades, whole-genome sequencing has revolutionized genetic research in all kingdoms, including fungi. More than 1000 fungal genomes have been submitted to sequence databases, mostly obtained through second generation short-read DNA sequencing. As a result, highly fragmented genome drafts have typically been obtained. However, with the emergence of third generation long-read DNA sequencing, the assembly challenge can be overcome and highly contiguous assemblies obtained. Such attractive results, however, are extremely dependent on the ability to extract highly purified high molecular weight (HMW) DNA. Extraction of such DNA is currently a significant challenge for all species with cell walls, not least fungi. In this study, four isolates of filamentous ascomycetes (*Apiospora pterospermum*, *Aspergillus* sp*. (subgen. Cremei*), *Aspergillus westerdijkiae*, and *Penicillium aurantiogriseum*) were used to develop extraction and purification methods that result in HMW DNA suitable for third generation sequencing. We have tested and propose two straightforward extraction methods based on treatment with either a commercial kit or traditional phenol-chloroform extraction both in combination with a single commercial purification method that result in high quality HMW DNA from filamentous ascomycetes. Our results demonstrated that using these DNA extraction methods and coverage, above 75 x of our haploid filamentous ascomycete fungal genomes result in complete and contiguous assemblies.

## Data Summary

**Impact Statement**

Sequencing high molecular weight (HMW) DNA by long read sequencing technologies facilitates the *de novo* assembly process and results in highly contiguous assemblies. However, generating HMW DNA is not straightforward from many organisms, including filamentous ascomycetes. In this work, we present two straightforward methods for extracting HMW DNA from ascomycete fungi and demonstrate that these methods can be effectively used in conjunction with established sequencing technology and bioinformatic analysis to generate highly contiguous genome assemblies from filamentous ascomycetes. Together with the increased flexibility of sequencing technologies that requires less specialized expertise and setup, this will contribute to increasing the accessibility of genome sequencing and analysis to many more laboratories. In turn, this will result in increased knowledge about fungal genetics for a much wider diversity of fungi.

The polished assemblies with maximum coverage for *Penicillium aurantiogriseum*, *Aspergillus westerdijkiae*, *Aspergillus* sp*. (subgen. Cremei*), and *Apiospora pterospermum* were uploaded to GenBank under the following accession number JAFCIW000000000, JAFBMQ000000000, JAFBMR000000000, and JAFBMP000000000, respectively. Unfiltered fastq files were uploaded to SRA for *Penicillium aurantiogriseum* (SRR13616996), *Aspergillus westerdijkiae* (SRR13616997), *Aspergillus* sp*. (subgen. Cremei*) (SRR13616995), and *Apiospora pterospermum* (SRR13616920).

## Introduction

Since the genome of *Saccharomyces cerevisiae* was released in 1996 [1], a number of fungal genome projects have been launched, notably the Fungal Genome Initiative [2] and 1000 Fungal Genomes Project [3]. During the last two decades, more than 1000 fungal genomes have been submitted to sequence databases, expanding the knowledge of the genetics, evolution, and diversity of fungi [4]. Most of these genomes were sequenced using short-read DNA (Illumina) sequencing [5–7]. Using short-read data for genome assembly typically leads to genome drafts composed of relatively short contigs [8, 9]. With the introduction of third generation long-read DNA sequencing, e.g. Oxford Nanopore Technologies (ONT) and Pacific Biosciences, a shift towards hybrid assemblies using both high accuracy short-reads and lower accuracy long-reads was observed in later years [10–12]. However, as raw read accuracy has increased [13, 14] and the cost of long-read DNA sequencing has decreased [15, 16], stand-alone long-read assemblies have emerged [17–19]. The principal advantage of using long-reads during the assembly process is that repetitive elements and complex structural variants can often be resolved to a greater extent than in assemblies generated from short-read sequencing. This leads to less ambiguous assemblies and the possibility of exploring previously inaccessible genomic features [9, 16].

Advantages of using ONT are that it only requires little initial capital investment in instruments and other facilities [20], is highly scalable [15], and portable [21]. ONT is therefore easy to incorporate in many laboratories. Having access to easy and cheap sequencing of many filamentous fungi has the potential to fundamentally change the ways filamentous fungi are studied and novel secondary metabolites are discovered. Instead of analysing spectra of the metabolites themselves, which is often inhibited by the inability to obtain growth conditions conducive to the production of the compound of interest, the whole biosynthesis potential might be predicted from the genome sequence itself and the candidate compounds produced in recombinant hosts [22, 23]. Furthermore, when creating deletion and overexpressing mutants, whole-genome sequencing can unambiguously document that the expected mutant is created without unintended secondary events. As a result, whole-genome sequencing is likely to become the future gold standard for verification of mutants, replacing the existing and less accurate standards using PCR and Southern Blotting only. Furthermore, ONT has, in principle, no upper limit to sequencing length, which essentially means that the read length distribution is determined by the extraction process and the fragmentation during library preparation. Importantly, the longer the raw reads obtained, the simpler downstream bioinformatics assembly processing becomes [9, 24]. However, extraction of high molecular weight (HMW) DNA from filamentous fungi is not trivial due to the cell walls, in particular their thickness and chemical complexity. Furthermore, the diversity of cell wall structure is challenging for developing general extraction methods that perform well across a range of fungi. Traditionally, in order to obtain high DNA yield, rather tough mechanical treatment during lysis is used to break the cell wall, but this negatively affects the size distribution of the DNA and is unsuitable for long-read sequencing [25, 26]. Recently, some protocols have emerged suggesting how to extract HMW fungal DNA for third generation sequencing. These primarily focus on CTAB and SDS for lysis [27–30]. However, documentation of robustness and resulting quality is sparse. Since DNA is very vulnerable to fragmentation during extraction, it is of paramount importance to choose an extraction method carefully.

In this study, we present two straightforward, versatile, and robust methods to extract and purify HMW DNA from four isolates of filamentous ascomycetes, both of which are suitable for ONT sequencing. From these, contiguous and accurate haploid assemblies were generated using a workflow of open-source bioinformatics software to benchmark the assembly outcomes when sequencing the extracted HMW DNA using ONT sequencing only.

## Methods

### Fungi

Three filamentous ascomycete strains (*Penicillium aurantiogriseum* (IBT 35659), *Aspergillus westerdijkiae* (IBT 35663), and *Aspergillus* sp*. (subgen. Cremei*) (IBT 35662)) were obtained from IBT Culture Collection of Fungi at the Technical University of Denmark (DTU, Denmark). The *Apiospora pterospermum* (CBS 123185) was obtained from Westerdijk Fungal Biodiversity Institute (Utrecht, The Netherlands). A 100 ml liquid yeast extract sucrose (YES) medium (20 g l^−1^ yeast extract (ThermoFisher), 150 g l^−1^ sucrose (VWR), 0.5 g l^−1^ MgSO4 ∙ 7 (Sigma-Aldrich), 0.016 g ZnSO4 ∙ 7 H2O (Sigma-Aldrich), and 0.005 g CuSO4 ∙ 7 H2O (Sigma-Aldrich)) was inoculated with five plugs (5×5 mm) of fresh fungi grown on solid YES-media. The fungi were grown at 25 °C for approximately 5 days in a circular shaker at 150 r.p.m. The mycelium from each fungus was harvested by filtering the liquid through a Miracloth (Millipore) and washed with 20 ml sterile Milli-Q water. The mycelium was lyophilized using a freeze-dryer overnight and subsequently ground in a mortar to a fine powder at room temperature.

### Evaluation of DNA extraction and purification methods

We initiated our investigation by an evaluation of six different combinations of extraction and purification methods: (1) Extraction and purification using DNeasy PowerSoil Kit (Qiagen), (2) Extraction using DNeasy PowerSoil Kit (Qiagen) and purification using phenol-chloroform, (3) Extraction and purification using phenol-chloroform only, (4) Extraction using phenol-chloroform and purification using AMPure beads XP (Beckman), (5) Extraction using phenol-chloroform and purification using QIAGEN Genomic-Tips 20 G^−1^, and 6) Extraction using Genomic Buffer Set (Qiagen) and purification using QIAGEN Genomic-Tips 20 G^−1^.

DNA extractions using DNeasy PowerSoil Kit (Qiagen) were performed on 25 mg lyophilized and ground mycelium according to the manufacturer’s protocol, including bead beating steps of 0 min, 1 min, 5 min, and 10 min. DNA extractions using phenol-chloroform were performed in four 2 ml Eppendorf tubes with 90 mg lyophilized and ground mycelium in each. The mycelium was carefully mixed with 1200 µl extraction buffer (100 mM tris-HCl, pH 8.0, 20 mM EDTA, 0.5 M NaCl, and 1 % SDS) and 700 µl phenol:chloroform:isoamyl alcohol (25 : 24 : 1) (Phenol:Chloroform Kit, pH 8, ThermoFisher) in each tube. Subsequently, the slurry was mixed by turning the tubes upside down until it was homogenized. The mixtures were incubated on a HulaMixer for 10 min at room temperature and subsequently centrifuged at 14 000 times gravity (× ***g***) for 5 min at room temperature. The aqueous layer in each of the four tubes was transferred to four new 2 ml Eppendorf tubes. Then 4 µl of RNase A (100 µg ml^−1^) (Qiagen) was added to each tube. The tubes were carefully turned upside down 10 times and incubated for 30 min at 50 °C. Phenol:chloroform:isoamyl alcohol (25 : 24 : 1) (Phenol:Chloroform Kit (pH 8), ThermoFisher) was added to the mixture in each tube in the ratio 1 : 1, and the tubes were carefully turned upside down 10 times and centrifuged at 14 000 × ***g*** for 5 min at room temperature. The aqueous layer from the four tubes was transferred to a 15 ml Falcon tube. DNA extraction using Genomic Buffer Set (Qiagen) was performed according to the manufacturer’s protocol with the following small modifications: (1) three 2 ml Eppendorf tubes with 25 mg ml^−1^ lyophilized and ground mycelium were used instead of cells directly from medium, (2) Lysing Enzymes from Trichoderma harzianum (Sigma-Aldrich) with a final concentration of 5.5 mg ml^−1^ was used instead of lyticase, (3) enzymatic degradation of the cell wall was performed at 37 °C for 1 h and cell lysis was performed at 50 °C for 2 h instead of the recommended time and temperature.

Purification using spin columns from the DNeasy PowerSoil Kit (Qiagen) was performed according to the manufacturer’s protocol. The DNA extracted and purified using phenol-chloroform only was precipitated by adding 0.1 vol of ammonium acetate (7.5 M) followed by 0.7 vol of isopropanol and carefully mixed by turning the tubes upside down until it was homogeneous. Following incubation for 30 min at room temperature, the sample was centrifuged for 30 min at 8000 × ***g***. The precipitated DNA was washed twice with 1 ml ice-cold ethanol (80%). The DNA pellet was dried for 30 s and dissolved in 65 µl tris-HCl buffer (10 mM, pH 8.5). The samples were mixed on a HulaMixer overnight at room temperature. Purification using AMPure beads XP was performed by adding equal amounts of DNA and AMPure beads XP solution (Beckman) and mixing it by flicking the tubes. The samples were incubated 2 min at room temperature. Beads were magnetized and the supernatant discarded. Subsequently, the beads were washed twice with 200 µl ethanol (80%) and dried for 30 s at room temperature, and the DNA was eluted in 65 µl tris-HCl (10 mM, pH 8.5). Finally, purification using QIAGEN Genomic-Tips 20 G^−1^ was performed according to the manufacturer’s protocol with the following minor modifications: the tips were washed four times and, during the precipitation step, the sample was incubated for 30 min at room temperature before centrifugation. Purification using QIAGEN Genomic-Tips 20 G^−1^ of the DNA extracted using phenol-chloroform was performed by mixing the sample with equal amounts of binding buffer (1600 mM guanidine HCl, 60 mM tris-HCl, pH 8.0, 60 mM EDTA, pH 8.0) prior to loading it on an equilibrated QIAGEN Genomic-Tip 20 G^−1^. In contrast, DNA extracted using Genomic Buffer Set was loaded directly on the QIAGEN Genomic-Tips 20 G^−1^. In both cases, the purified DNA was incubated on a HulaMixer overnight at room temperature to allow it to be resuspended.

Quality control of the purified DNA was performed using NanoDrop One (ThermoFisher), Qubit 3.0 (Invitrogen) with Qubit dsDNA HS Assay Kit, and 2200 TapeStation (Agilent) with Genomic DNA ScreenTape Analysis according to the manufacturers’ instructions.

### Removal of small fragments from the extracted DNA

Circulomics Short Read Eliminator XS was used to remove small fragments from the DNA preparations according to the manufacturer’s protocol. Quality control of the DNA was performed as described above.

### Library preparation and sequencing

The selected samples were sequenced on a single R9.4.1 flow cell using the Native barcoding genomic DNA (EXP-NBD104, EXPNBD114, and SQK-LSK109) protocol from ONT (Oxford, UK).

### Genome assembly

The raw reads were basecalled, and demultiplexed, and adapters were removed using Guppy version 3.4.4 (Oxford Nanopore Technologies, England) in GPU mode using the dna_r9.4.1_450bps_hac.cfg model. The basecalled reads were subsequently filtered to a minimum length of 10 kb and a minimum quality of 80 (Q7) using Filtlong version 0.2.0 [31]. NanoPlot version 1.24.0 was used to evaluate the resulting reads [32]. Subsets (10x-, 25x-, 50x-, 75x- and 100x-coverage) were extracted randomly using seqtk version 1.3 [33]. Overlaps between the filtered reads were mapped using Minimap2 version 2.15 [34] with default settings, and the assemblies were created as haploids using Miniasm version 0.3 [35] with default settings. Reads were mapped back to the newly created assembly using Minimap2 version 2.15 with default settings. The assembly was subsequently polished in three rounds; first using Racon version 1.3.3 [36] with default settings, then Medaka version 0.11.5 [37] with default settings followed by a final round of Medaka version 0.11.5 with default settings.

### Genome assembly evaluation

Assembly completeness was evaluated using the Ascomycota BUSCO dataset version nine and Benchmarking Universal Single-Copy Orthologs (BUSCO) version 3.0.2 [38]. Random indel errors and indel errors in homopolymeric regions were investigated in the different subsets by aligning three BUSCO genes (EOG092D0072, EOG92D005G, and EOG092D00H) observed in a single copy in all the assemblies in CLC Genomics Workbench version 20.0 (QIAGEN, Århus). The filtered reads were mapped back to the newly created assemblies polished with Racon and two rounds of Medaka using Minimap2 version 2.15 [34]. The average mapping completeness was calculated from the resulting .sam file using a custom bash command (Supplementary note 1). In short, the command extracts the number of ‘softclipped’ bases at each end of every read and divides it with the total length of the read. Then the average of this ratio across all reads is calculated. Read mapping completeness can then be calculated by subtracting this value from 1. Tandem repeat elements were identified using TandemRepeat Finder (TRF) version 4.09 [39]. Repeat sequences were identified using RepeatMasker version 4.1.2 with eukaryote as species [40]. Noncoding RNAs were predicted using Barrnap version 0.9 [41] and tRNA-scan version 2.0.5 [42]. Blobtools2 from Blobtoolkit version 3.0.0 in combination with NCBI nucleotid database release 234 was used to screen for contaminations in the final assemblies [43]. The mapped reads were visualized in CLC to confirm uniform read coverage distribution. Telomeric regions were found based on the fungal telomeric repeat sequence of TTAGGG/CCCTAA using the motif search in CLC Genomics Workbench and visualized by a sliding window analysis (bin size 100 b and step size 25 b). Only 100 % identity matches were included in the analysis. All figures were visualized in R version 3.6.2 in RStudio version 1.3.1093 [44] using ggplot2 version 3.3.3 [45].

## Results

### Evaluation of DNA extraction methods

The essential criteria for successful sequencing using ONT are the purity and size distribution of the extracted DNA. Six different combinations of DNA extraction and purification methods were therefore examined using four filamentous ascomycetes (*Penicillium aurantiogriseum*, *Aspergillus westerdijkiae, Aspergillus* sp*. (subgen. Cremei*), and *Apiospora pterospermum*) in order to determine suitable methods for extraction and purification of DNA. ONT recommends an A260/280 ratio of 1.8, an A260/230 ratio of 2.0–2.2, and a ratio between the concentration measured on the NanoDrop and the concentration measured on the Qubit (NanoDrop/Qubit ratio) of 1.0–1.5. It is, however, also essential to assess the fragment length distribution of the purified DNA, which should be devoid of small DNA fragments, as well as the amount of the extracted DNA (preferably >1.5 µg).

**Fig. 1.**
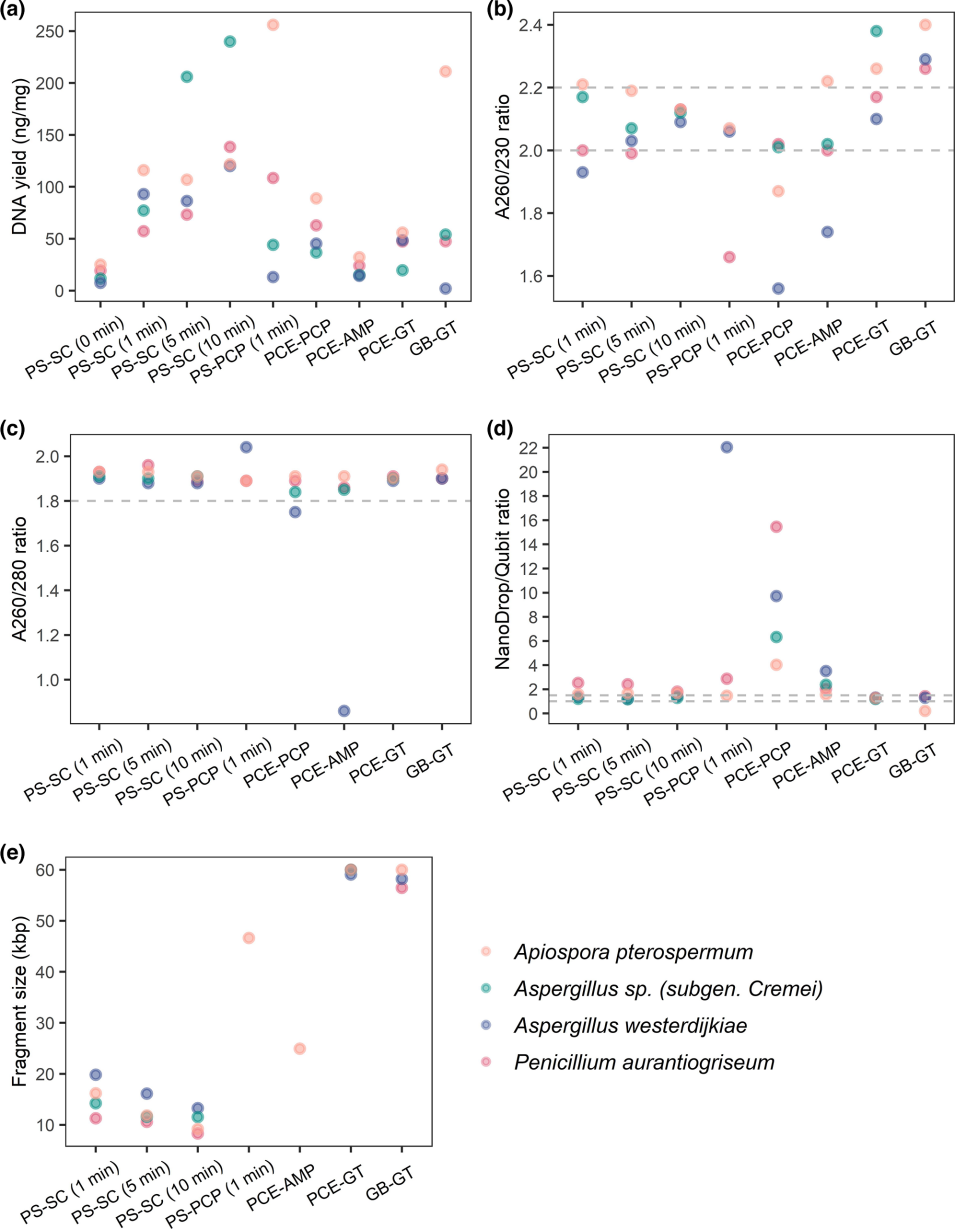
The effect of six different combinations of DNA extraction and purification methods on four fungi (*Apiospora pterospermum*, *Aspergillus westerdijkiae*, *Penicillium aurantiogriseum*, and *Aspergillus* sp. (*subgen*. *Cremei*)). PS-SC; QIAGEN DNeasy PowerSoil Kit with bead beating for indicated time periods followed by purification using Spin columns from QIAGEN DNeasy PowerSoil Kit. PS-PCP; QIAGEN DNeasy PowerSoil Kit with bead beating for 1 min followed by phenol-chloroform purification. PCE-PCP; only phenol-chloroform extraction and purification. PCE-AMP; phenol-chloroform extraction followed by AMPure XP beads purification. PCE-GT; phenol-chloroform extraction and QIAGEN Genomic-Tips 20 G^−1^ purification. GB-GT; QIAGEN Genomic Buffer Set and QIAGEN Genomic-Tips 20 G^−1^ purification. (a) DNA yield (ng DNA/mg dry cell mass). (b) A260/230 and (c) A260/280 ratio; all samples with a concentration below 20 ng µl^−1^ were excluded from the figure. (d) Ratio of concentration determinations (UV-abs/Fluorescence); samples with a concentration above 20 ng µl^−1^ were measured only. (e) Mean fragment size (kbp) of the extracted DNA; samples that complied with the listed criteria were measured only. Hatched grey lines in b–d indicate recommended intervals.

The results from the six combinations of extraction and purification methods on the four filamentous ascomycetes are shown in Fig. 1. Nearly all methods yielded acceptable A260/A280 ratios (Fig. 1c) and all, with the exception of DNA extracted and purified with phenol-chloroform, yielded acceptable NanoDrop/Qubit ratios (Fig. 1d). The very low A260/A280 ratio of this sample is probably caused by the presence of visible insoluble material, likely a carbohydrate polymer. In general, the combination of bead beating and DNeasy PowerSoil Kit yielded the highest amounts of extracted DNA (Fig. 1a) compared to the other methods, but increased fragmentation of the DNA was observed (Fig. 1e). Taken together, Genomic Buffer Set in combination with Genomic-Tips 20 G^−1^ and phenol-chloroform in combination with Genomic-Tips 20 G^−1^ were the overall best methods examined. A detailed protocol of these methods is described in Supplementary note 2. In the case of *Aspergillus* sp*. (subgen. Cremei),* however, only a relatively low amount (2.04 ng DNA/mg dry cell mass) of DNA was extracted using Genomic Buffer Set in combination with Genomic-Tips 20 G^−1^. This could be due to poor degradation of the cell (Fig. 1a) since a disproportionally higher yield (48.57 ng DNA/mg dry cell mass) was observed using phenol-chloroform in combination with Genomic-Tips 20 G^−1^ for this fungus. The degradation of the cell wall was digested with varying efficiency, probably due to different chemical composition and thickness of the cell walls. From this, it can also be assessed that one cannot except a extraction method can be applied for every species within a genus. Noteworthy, the amount of fungal material required is higher for the phenol-chloroform method (see Methods for details) compared to the Genomic Buffer Set, which in some cases might speak against the use of the former method.

### Filtering of fragments and reads improve the input for assembly

For sequencing, we chose to use the DNA extracted using Genomic Buffer Set for *Penicillium aurantiogriseum*, *Aspergillus westerdijkiae,* and *Apiospora pterospermum* and phenol-chloroform extraction for *Aspergillus* sp*. (subgen. Cremei*). All purifications were conducted using the Genomic-Tips 20 G^−1^. Before sequencing, small fragments (<10 kbp) were removed from the samples using Circulomics Short Read Eliminator XS. This increases the DNA integrity number (DIN) from 6.6 to 9.4 to 8.9–9.8 (Supplementary note 3).

After sequencing, basecalling and demultiplexing, an average of 5.71 (±0.81) Gb and an average of 562 690 (±334 124) reads were obtained (Table 1). Following demultiplexing, 11 % of the reads were unclassified reads. The mean quality of the unclassified reads was significantly lower (Q6.7 compared to Q11.9). Therefore, the majority of the unclassified reads would not have been included in the assembly process even if they have been assigned to a certain sample. Following quality and length filtering, 4.40 (±1.1) Gb and an average of 180 520 (±48 490) reads were retained. N50 of the read distribution, which is the minimum read length needed to cover 50 % of the read distribution, ranged from 13.10 to 29.92 kb before filtering, and 24.85–32.87 kb after filtering. The mean basecalling quality of reads was similar before and after filtering. Interestingly, the N50 of *Aspergillus* sp*. (subgen. Cremei*) is significantly lower than the other samples, and since this sample was the only sample extracted using phenol-chloroform and purified using Genomic-Tips 20 G^−1^, this suggests that this method leads to higher fragmentation of the DNA. However, other samples extracted and purified in the same way by us (data not included in this study) produced similar N50 as samples extracted with Genomic Buffer Set and purified with Genomic-Tips 20 G^−1^, thus not supporting this general conclusion.

**Table 1.** Summary of sequencing statistics. Filtering was conducted by Filtlong version 0.2 using a minimum read length of 10 kb and minimum quality of Q7

Before filtering				
	*Penicillium aurantiogriseum*	*Aspergillus westerdijkiae*	*Aspergillus* sp*.* *(subgen. Cremei*)	*Apiospora pterospermum*
No. of bases (Gb)	5.57	5.31	5.06	6.89
No. of reads	397 011	304 966	1 051 356	497 426
N50 (kb)	27.12	29.92	13.10	21.05
Mean read quality	Q12.0	Q12.0	Q11.8	Q11.9
**After filtering**				
No. of bases (Gb)	4.57	4.67	2.94	5.60
No. of reads	169 574	168 878	134 614	249 012
N50 (kb)	32.61	32.87	24.85	24.70
Mean read quality	Q12.1	Q12.2	Q12.1	Q12.0

### Genome assembly

The fungal strains used in this study are all haploid and expected to harbour low genome complexity (e.g. small percentage of repetitive elements) [5, 46–48]. From previous studies, the expected size of the genomes are 48 Mb for *Apiospora pterospermum* [46], between 29–36 Mb for *Aspergillus westerdijkiae* and *Aspergillus* sp*. (subgen. Cremei*) [49], and between 28.3–40.9 Mb for *Penicillium aurantiogriseum* [50].

**Fig. 2.**
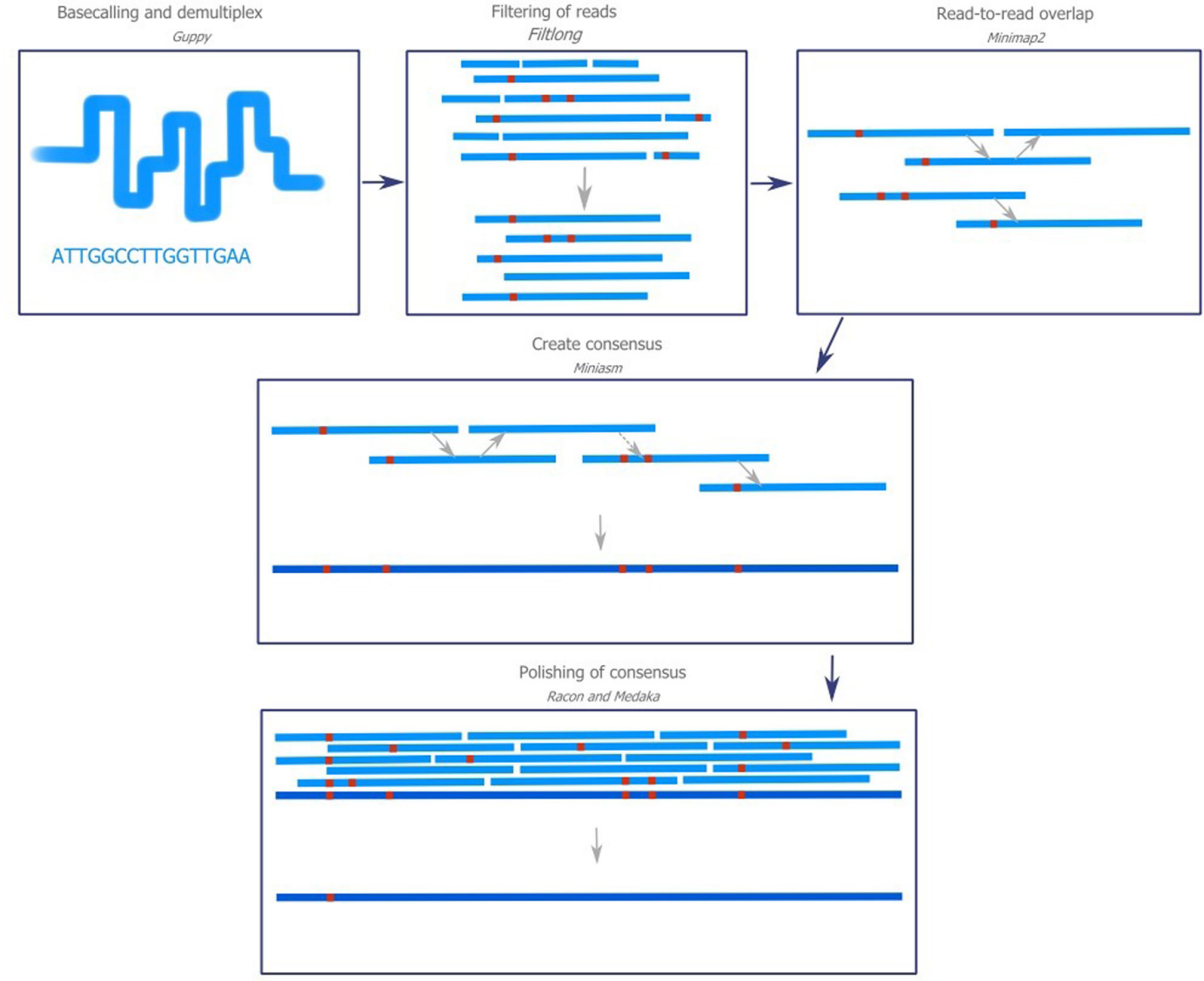
Overview of the bioinformatics workflow. Programs used in the different steps are denoted with italics. Nucleotide mismatches are marked as red squares. Statistics with regard to the four fungi analysed in this study can be found in Table 1. Guppy is used for basecalling and demultiplexing the raw reads. Reads ≥10 kb and quality threshold ≥80 are removed using Filtlong. After filtering of the reads, an assembly can be created using the assembler Miniasm but it requires read-to-read overlap. To perform this, the program Minimap2 is used. Polishing of the consensus is performed using the two programs Racon and Medaka since these have shown to increase the completeness significantly. A second round of Medaka can be used to polish the assembly.

*De novo* assemblies for the filamentous ascomycetes were created using open-source bioinformatics software (Fig. 2). The software chosen in this workflow is an example of software, which can be used in an assembly process. It is possible that other software components can create similar or even slightly better results. The purpose of this part of the study is not to document the performance of different software, but to show a workflow that is sufficient to achieve high quality genome drafts. These tools were chosen because they performed comparable to more computer-intensive options [51–53] as well as during testing in our hands. However, software and algorithms for genome assembly and analysis are under rapid development [54]. It is therefore recommended to update the used software to the newest version and test substitute components for future better options.

This bioinformatics workflow uses the assembler tool Miniasm. This assembler does not include a sequence error correction step. As a result, the initial consensus accuracy is the same as sequencing accuracy [35]. Hence, polishing of the assembly is therefore required to improve consensus accuracy and completeness. Racon and Medaka are currently some of the most popular polishing tools for ONT data [36, 37]. Polishing of the assembly has also been shown to decrease homopolymer errors, which is a frequent type of error in ONT sequencing and typically leads to small indels in the final assembled consensus [17]. Small indels are particularly disruptive when translating predicted gene sequences into protein sequences because they frequently lead to frameshifts. Hence, correction of this type of error is particularly important. Therefore, Racon and a combination of Racon and Medaka were tested (Table 2). Medaka was not tested individually since the tool was developed to correct assemblies which already have been polished with Racon [37]. Because *Apiospora pterospermum* has not previously been sequenced and only related strains of *Penicillium aurantiogriseum* [55], *Aspergillus westerdijkiae* (NCBI accession number ASM130734v1), and *Aspergillus* sp*. (subgen. Cremei*) (NCBI accession number Aspwe1) are available, direct comparison of the obtained assemblies to an established reference could not be conducted. Consensus accuracy was therefore not investigated in these assemblies, but other studies have found an average identity of >99.5 % when using a similar approach (Miniasm and Racon) [51, 52]. Instead, assembly evaluation was based on the number of contigs, assembly size of the draft, BUSCO analysis, and N99 (Table 2). N99 of the assemblies is a measure of contiguity. It is the minimum contig length needed to cover 99 % of the genome. A high N99 means less fragmentation of the assembly. Furthermore, the assemblies were also evaluated with regard to repetitive elements, rRNA and tRNA genes, amount of unmapped reads, and coverage distribution.

**Table 2.** Overview of statistics of polishing steps. See Methods for details. C (%) and F (%) denotes BUSCO completeness in percent and fragmented BUSCOs in percent, respectively. A total of 1315 genes were considered in the BUSCO analyses

	Coverage	Polishing steps	no. of contigs	N99 (b)	Genome size (Mb)	C (%)	F (%)
*Apiospora pterospermum*	125 x	None	18	394 867	44.2	2.2	7.2
*Apiospora pterospermum*	125 x	Racon	18	398 398	44.7	90	5.5
*Apiospora pterospermum*	125 x	Racon+Medaka	17	398 846	44.7	98	0.8
*Apiospora pterospermum*	125 x	Racon+MedakaX2	17	398 820	44.7	98	0.8
*Aspergillus westerdijkiae*	130 x	None	11	2 708 532	35.7	1.6	8.3
*Aspergillus westerdijkiae*	130 x	Racon	10	2 734 490	36	88.3	5.8
*Aspergillus westerdijkiae*	130 x	Racon+Medaka	10	2 736 658	36	98	0.7
*Aspergillus westerdijkiae*	130 x	Racon+MedakaX2	10	2 736 562	36	97.9	0.7
*Penicillium aurantiogriseum*	139 x	None	8	4 594 295	32.4	1.3	9.4
*Penicillium aurantiogriseum*	139 x	Racon	8	4 638 995	32.8	90.8	4.9
*Penicillium aurantiogriseum*	139 x	Racon+Medaka	8	4 642 496	32.8	97.5	0.8
*Penicillium aurantiogriseum*	139 x	Racon+MedakaX2	8	4 642 389	32.8	97.5	1.1
*Aspergillus* sp*. (subgen. Cremei*)	91 x	None	11	2 636 682	31.9	1.1	7.6
*Aspergillus* sp*. (subgen. Cremei*)	91 x	Racon	11	2 655 487	32.2	87.1	6.9
*Aspergillus* sp*. (subgen. Cremei*)	91 x	Racon+Medaka	11	2 657 265	32.2	97.6	0.5
*Aspergillus* sp*. (subgen. Cremei*)	91 x	Racon+MedakaX2	11	2 657 265	32.2	97.8	0.5

In two cases, the number of contigs was unchanged following polishing, but in the other two cases, *Aspergillus westerdijkiae* (130 x coverage) and *Apiospora pterospermum* (125 x coverage), the number of contigs was reduced by one (Table 2). N99 and total genome size were stable during polishing. Completeness, as assessed by BUSCO analysis, increased markedly after the first round of polishing from 1.1–3 % to 87.7–90.8 % for all the assemblies. The second round of polishing increased completeness with a further 6.7–10.5 %. The final BUSCO completeness for all assemblies was approximately 98 %.

Furthermore, the sizes of the assemblies were as expected and comparable to the literature [46, 49, 50] (Table 2). The assemblies contain 6.38–7.79% repetitive elements where especially retroelements and tandem repeats constitute the largest shares. Additionally, 0.13–0.17 % of the assemblies were rRNA and tRNA genes (Supplementary note 4). This is consistent with what has been observed previously [5, 46–48].

Only a small percentage of unmapped reads was observed (0.03–0.44 %), indicating that the created assemblies contained most genomic features. Almost all contigs in the four assemblies showed a uniform distribution of coverage. However, an increased coverage was observed in a region at one end of contig 3, 6, 5, 4 from the assembly of *Apiospora pterospermum*, *Aspergillus westerdijkiae, Penicillium aurantiogriseum,* and *Aspergillus* sp*. (subgen. Cremei*), respectively. These regions were identified to contain rRNA genes, which are frequently observed as repetitive elements in genome assemblies, in all cases.

In general, it is possible to produce contiguous assemblies with high completeness, when using DNA extracted and purified using the two methods shown in this study and using ONT sequencing only. It is, however, important to include polishing of the consensus when creating the fungal assemblies using Miniasm. The third round of polishing, however, only provides a minor increase in completeness and could therefore be omitted if computational time is a limiting resource. These assemblies were created from reads basecalled using Guppy version 3. It is possible that using the newest version of Guppy will increase the assembly quality slightly, since this basecaller is under constant development [56].

### Sequencing depth above 75x coverage is necessary for high quality assemblies

**Fig. 3.**
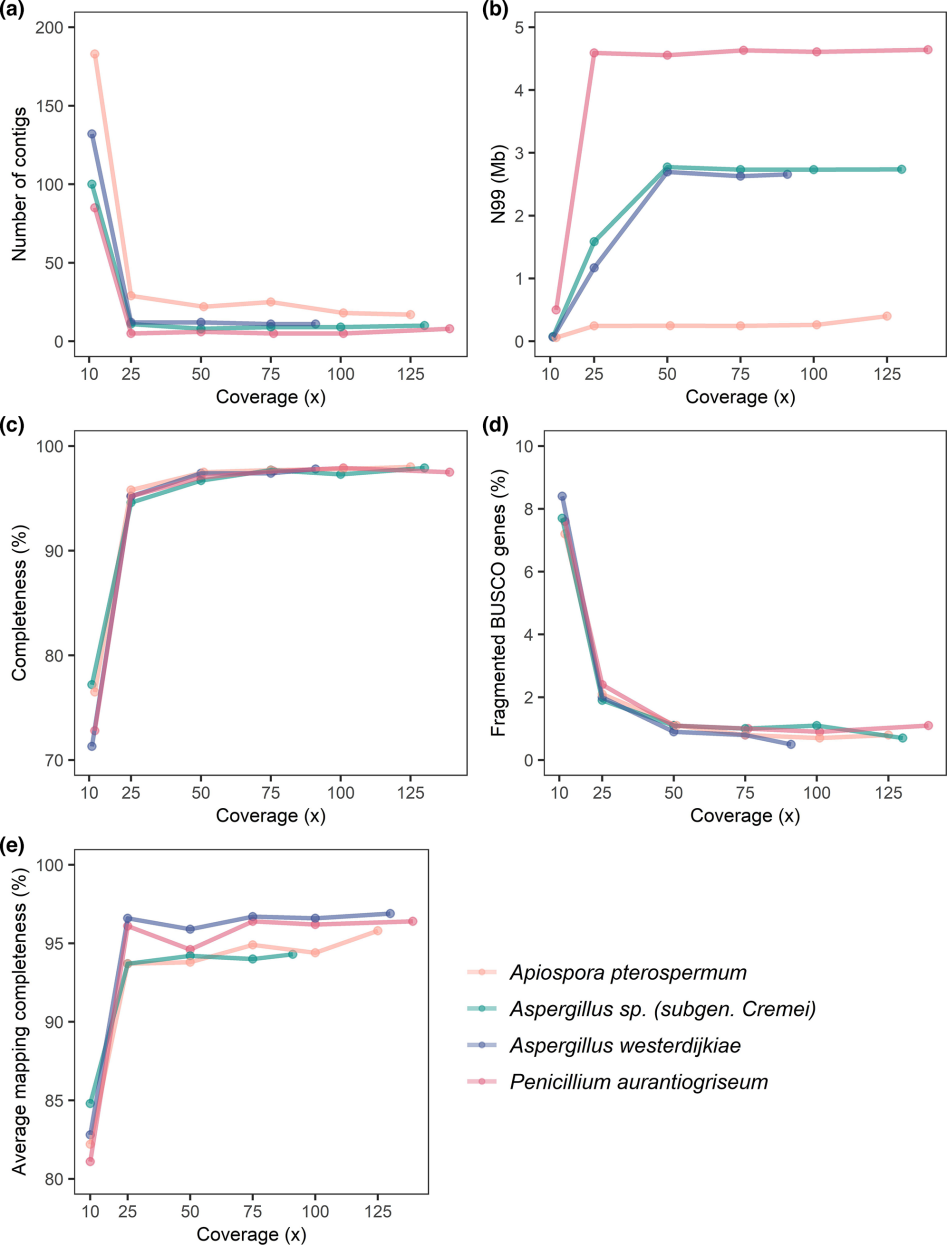
Influence of coverage on the assemblies. (a) Number of contigs as a function of coverage. (b) N99 as a function of coverage. (c) Completeness as a function of coverage. Completeness was assessed from BUSCO analysis. (d) Number of fragmented BUSCO genes as a function of coverage. (e) Average mapping completeness (%): the average of the fraction of any read that maps to the assembly across all reads.

The influence of sequence coverage on the genome drafts was investigated by creating assemblies on randomly subsampled pools of reads. A way to evaluate the influence is to explore the contiguity of the assemblies. A high contiguity is attractive since it can approximate full chromosome models for these fungi. The contiguity was increased by a factor of 10 when increasing coverage from 10 to 25x (Fig. 3a). Increasing coverage further had little effect on contiguity. In two cases, however, increasing the coverage to more than 100 x led to slightly reduced contiguity (*Penicillium aurantiogriseum* (5 to 8 contigs) and *Aspergillus westerdijkiae* (9 to 10 contigs)). This suggests that excessive coverage might lead to artefactual contigs that should be identified and removed post-assembly. Often simply observing the read coverage of contigs is sufficient to identify these artefacts. Typically, an assembly contains (1) few small contigs with very high coverage often represent mtDNA, shorter contigs containing repeat elements or ribosomal arrays, (2) a group of larger contigs with similar read coverage, likely representing nuclear DNA and thereby ‘true contigs’, and (3) some contigs with lower read coverage, which are often but not always to represent artefactual contigs. This is, however, dependent on the complexity of the genome, and therefore not always the case. In this study, we have found that using a threshold of read coverage of 67 % of the mean read coverage of the plateau of the ‘true contigs’ is a useful and simple cutoff measure. This indeed is a naive approach, which may not be meaningful in other datasets with different genome complexity. It could be hypothesized that low coverage contigs may be due to contaminations or even symbionts. However, all contigs observed in the four assemblies were assigned to Ascomycota origin according to blobtools2 (data not shown), suggesting that this is not the case.

We found that the contiguty in terms of N99 for all assemblies reached a plateau at 25 x coverage or 50 x coverage (Fig. 3b). Furthermore, the assemblies were evaluated by analysing the completeness of gene annotation of the assemblies using BUSCO analysis (Fig. 3c and Supplementary note 5), as well as, the average mapping completeness of sequenced reads, thus assessing completeness beyond gene annotation (Fig. 3e). Including sequencing data to 50 x coverage leads to increased BUSCO and average mapping completeness, whereas fragmented BUSCO genes decreased (Fig. 3d). Additional sequencing data has little effect.

**Fig. 4.**
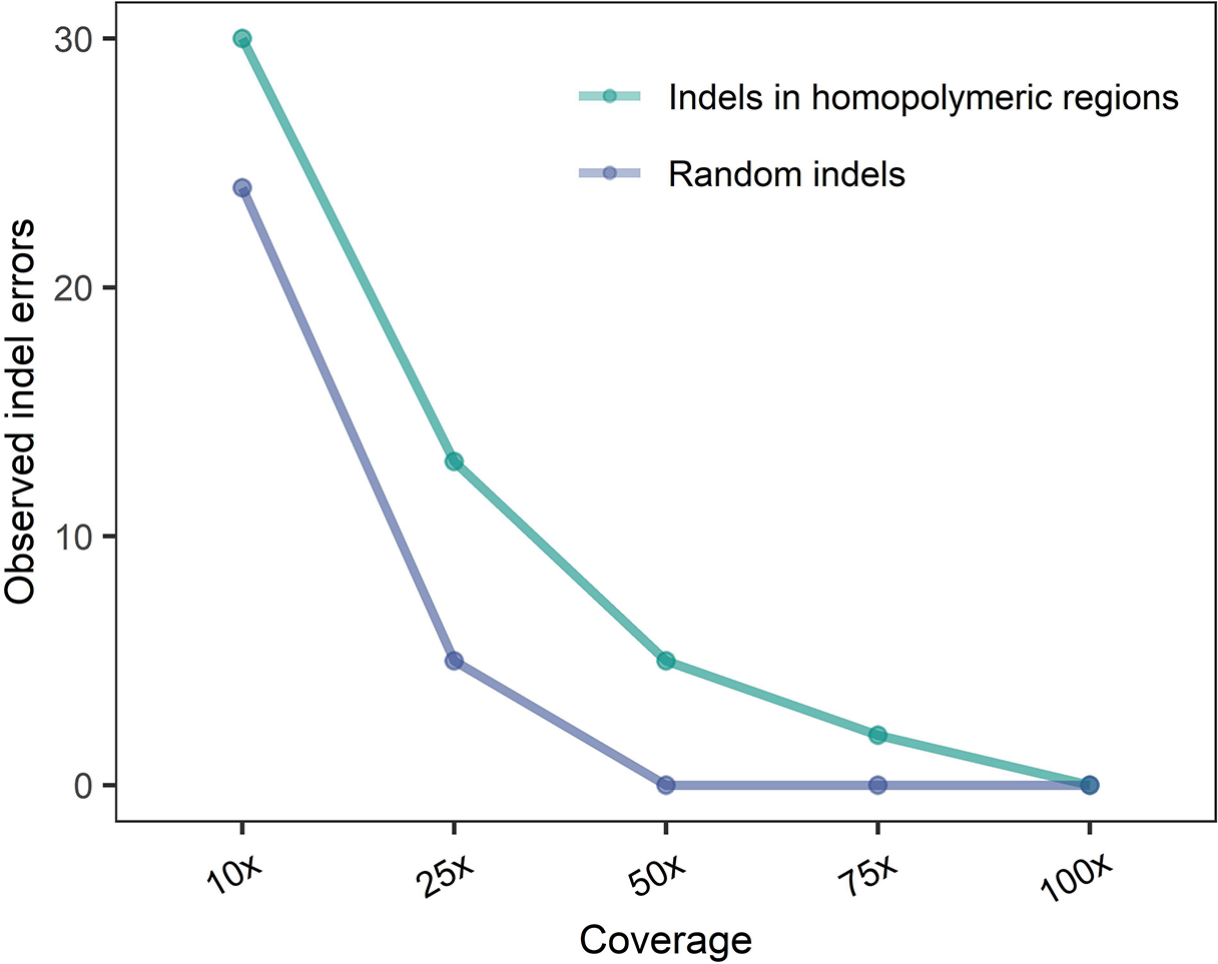
Counts of random indel errors and homopolymeric indel errors in assemblies polished with Racon and two rounds of Medaka at different coverage levels for the three BUSCO genes EOG092D0072, EOG92D005G, and EOG092D00. The sum of indel counts for all three genes is shown. The data point for coverage 10x – 75x is made with data from the four assemblies, whereas the data point for 100 x is only represented by data from three assemblies, since *Aspergillus* sp. (subgen. *Cremei*) was only sequenced to maximum of 91 x coverages.

Indels in homopolymeric regions and, to a lesser extent, indels outside homopolymeric regions are recognized as the main accuracy problem when sequencing using ONT [17]. To this end, three BUSCO genes (EOG092D0072, EOG92D005G, and EOG092D00H) present in all the assemblies were manually inspected for indels. Indeed, indels were observed within the genes from assemblies with low coverages (Fig. 4). However, when increasing the sequencing depth, the indel errors decrease. When coverage was increased to above 50 x, indels outside homopolymeric regions were not observed in the three genes investigated and at 100 x coverage, no indels in homopolymeric regions either were observed in the three genes. We do not infer from this sample analysis that no indel errors exist in the entire assembly, simply that the frequency of such errors was reduced markedly by ensuring a coverage of above 75 x. We believe that the best choice to decrease this even further is to correct the consensus accuracy by using high accuracy short-read data if needed.

In summary, the best assemblies were obtained using 75 x coverage since these assemblies were stable in terms of completeness, continuity, average mapping completeness, and indel errors. Assemblies created using a coverage above 50 x also perform well in our analysis. Thus, some researchers may choose to sequence more fungi with decreased coverage but this comes with an increased number of indels in homopylomeric regions.

### Telomeric regions can be found on both end of several contigs

To investigate to what extent the assemblies represent chromosome-level contigs, a sliding window search for telomeric repeats was performed for all contigs except those of mtDNA or contigs exclusively containing rRNA. The expected result for a chromosome is to find an increased number of telomere repeats at either end of the chromosome and few within chromosomes. The telomeres in many filamentous fungi consist of (TTAGGG)_n_ [57–61].

From this search, it is clear that some contigs from all four fungi, regardless of coverage above 50 x, have telomeric regions at both ends of the contigs judging by the number of the increased repeats at the ends (Table 3 and Supplementary note 6). This indicates that these contigs are chromosome-level models. On the other hand, no fungi had telomeric repeats at both ends of all large contigs, and many contigs with only one or no telomeric region were observed, especially in the case of *Apiospora pterospermum*. By and large, the sizes of the contigs do not determine if telomeric repeats are found at both ends since the size of contigs with both telomeric repeats is not unambiguously different from contigs with a single or no telomeric repeats (see Supplementary note 6). This analysis underscores that, while these assemblies are indeed of high quality, they are not perfect representations of chromosomes, and additional experiments must be conducted to provide finished assemblies.

**Table 3.** Overview of the telomeric regions in the different assemblies made for the four fungi all polished with Racon and two rounds of Medaka

	Coverage	Total no. of telomeric regions	no. of contigs with telomeric region at both ends	no. of contigs with telomeric region at one end	no. of contigs with no telomeric region*
*Apiospora pterospermum*	125 x	8	2	4	8
	100 x	10	1	8	6
	75 x	8	2	4	16
	50 x	12	1	10	8
*Aspergillus westerdijkiae*	130 x	7	1	5	2
	100 x	6	2	2	4
	75 x	7	2	3	3
	50 x	6	1	4	4
*Penicillium aurantiogriseum*	139 x	6	2	2	0
	100 x	5	1	3	0
	75 x	6	2	2	0
	50 x	6	2	2	0
*Aspergillus* sp. (subgen. *Cremei*)	91 x	9	3	3	2
	75 x	6	2	2	4
	50 x	6	1	4	2

*Contigs comprising mtDNA or exclusively rRNA genes are not included in the analysis.

## Conclusions

In this study, we have provided evidence to support the usefulness of two robust and versatile sets of DNA extraction and purification methods that are suitable for long-read sequencing. A bioinformatics workflow consisting of open-source software was devised and used to demonstrate the usability of the DNA extraction and purification methods in regards to producing high quality assemblies for the four ascomycete filamentous fungi used in this study. The key parameters for success are to extract pure HMW DNA without trace amounts of smaller fragments and have a sequence coverage above 75 x. This is compatible with multiplexing four genomes (~36 Mbp) in a single R9.4.1 flow cell and thus is not cost prohibitive for most laboratories.

## Supplementary Data

Supplementary material 1
