# Supplementary material for: High molecular weight DNA extraction methods lead to high quality filamentous ascomycete fungal genome assemblies using Oxford Nanopore sequencing
Source: Microb Genom. 2022 Apr 19;8(4):000816. doi: 10.1099/mgen.0.000816 (PMC9453082; doi:10.1099/mgen.0.000816)
Supplement: Supplementary material 1 [file mgen-8-0816-s001.pdf]

Supplementary note 1

awk -F"\t" '{print $6}' mapped_maybe.sam | sed -e 's/M/M|/g' -e 's/D/D|/g' -e 's/I/I|/g' -e 's/S/S|/g' -e 's/H/H|/g' -e 's/\=/\=|/g' -e 's/X/X|/g' | awk -F"|" -vOFS='\t' '{if(NF == 0) {print $1} else {print $1, $(NF-1)}}' | sed -e 's/\w*M//g' -e 's/\w*D//g' -e 's/\w*i//g' -e 's/\w*H//g' -e 's/\w*=//g' -e 's/\w*X//g' | sed 's/S//g' | awk '{for(i=t=0;i<=NF;i++){t+=$i}; print t}' > SCbases.tmp; awk -F"\t" '{print $6}' mapped_maybe.sam | awk -F"M|D|I|S|H|=|X" -vOFS='\t' '{for(i=t=0;i<=NF;i++){t+=$i}; print t}' > TOTbases.tmp; paste SCbases.tmp TOTbases.tmp | awk '{if($2>0) {print $1, $2, $1/$2}}' > ReadmappingCompleteness.tsv; awk '{sum+=$3}; END {print "Average read mapping completeness: ",sum/NR}' ReadmappingCompleteness.tsv

Supplementary note 2: Laboratory protocol

Materials

Reagents

Product	Supplier	Catalog number
Bacto^{TM} Yeast Extract	ThermoFisher	212750
Sucrose	VWR	27483.465
MgSO4 · 7 H2O	Sigma-Aldrich	63140-1KG
Agar	VWR	20768.292
ZnSO4 · 7 H2O	Sigma-Aldrich	Z0251-100G
CuSO4 · 7 H2O	Sigma-Aldrich	F8048-250G
Tris-HCl	Sigma-Aldrich	9310-500GM
EDTA	Sigma-Aldrich	EDS-500G
NaCl	VWR	BDH9286-12KG
SDS BioChemica	AppliChem	A2572,0250
Guanidine HCl	Sigma-Aldrich	G3272-500G
Phenol:Chloroform Kit (pH 8)	ThermoFisher	17909
RNase A (100 µg/ml)	Qiagen	19101
Isopropanol BioUltra	Sigma-Aldrich	59304-1L-F
Ethanol absolute	VWR	20821.310
β-mercaptoethanol	Sigma-Aldrich	8057400250
Proteinase K	Qiagen	19133
Lysing Enzymes from *Trichoderma harzianum*	Sigma-Aldrich	L1412-10G
Blunt/TA Ligase Master Mix	New England Biolabs	M0367
NEBNext® Quick Ligation Module	New England Biolabs	E6056
NEBNext® FFPE DNA Repair Mix	New England Biolabs	M6630
NEBNext® Ultra™ II End Repair/dA-Tailing Module	New England Biolabs	E7546
Agencourt AMPure XP beads	Beckman	A63881

Media, buffers and solutions

Trace solution for YES medium: dissolve 1.6 g $ZnSO_4 \cdot 7\ H_2O$ and 0.5 g $CuSO_4 \cdot 7\ H_2O$ in 100 mL Milli-Q.

Liquid YES-medium: dissolve 20 g yeast extract, 150 g sucrose, and 0.5 g $MgSO_4 \cdot 7\ H_2O$ in 800 mL demineralized water. Add 1 mL trace solution and fill up to 1000 mL with demineralized water and autoclave the solution.

Solid YES-medium: dissolve 20 g yeast extract, 150 g sucrose, 0.5 g $MgSO_4 \cdot 7\ H_2O$, and 20 g agar in 800 mL demineralized water. Add 1 mL trace solution and fill up to 1000 mL with demineralized water and autoclave the solution. Pour the warm solution into Petri dishes.

Extraction buffer: 100 mM tris-HCl pH 8.0, 20 mM EDTA, 0.5 M NaCl, and 1 % SDS. The buffer is prepared using Milli-Q (see Note 1).

Binding buffer: 1600 mM guanidine HCl, 60 mM tris-HCl (pH 8.0), and 60 mM EDTA (pH 8.0). Prepare the buffer using Milli-Q.

Tris-HCl buffer: dissolve 0.121 g tris-HCl in 80 mL Milli-Q. Adjust the pH to 8.5. Adjust the volume to 100 mL.

Kits

Product	Supplier	Catalog number
QIAGEN Genomic-Tips 20/G	Qiagen	10223
Genomic Buffer Set	Qiagen	19060
Short Read Eliminator XS	Circulomics	SKU SS-100-121-01
Ligation Sequencing Kit	ONT	SQK-LSK109
Flow Cell Priming Kit	ONT	EXP-FLP002
Optional: Native Barcoding Expansion 1-12 (PCR-free)	ONT	EXP-NBD104
Qubit™ dsDNA HS Assay Kit	ThermoFisher	Q32854
Genomic DNA ScreenTape	Agilent	5067-5365
Genomic DNA Reagents	Agilent	5067-5366

Protocol

Culturing and harvesting of fungi

1. Inoculate the fungi on solid YES-plates, and grow them between 7-14 days at 25°C depending on the growth rate of the species.
2. Inoculate a 100 mL liquid YES-media with five plugs (5x5 mm) of the fungi from solid YES-media. Incubate the fungi at 25°C for approximately 5 days in a circular shaker at 150 rpm.
3. Harvest mycelium by filtering through Miracloth. Wash with 20 mL sterile Milli-Q water. Lyophilize the mycelium overnight, and subsequently grind to a fine powder at room temperature.

Extraction and purification of HMW DNA

Use either of the methods described below. In some cases, the cell wall of some fungi is ineffectively degraded by Lysing Enzyme from *Trichoderma Harzianum* and thus phenol-chloroform extraction is the better choice. However, the input requirement is ~5x larger for this method, which in some cases may be prohibitive. Regardless of the extraction method chosen, it is crucial to handle the DNA carefully and not vortex or use extensive pipetting to mix the sample, but instead mix by turning upside down and/or flicking the tubes when indicated.

Extraction of HMW DNA using phenol-chloroform

1. Transfer 90 mg of lyophilized and ground mycelium to each of four 2 mL Eppendorf tubes, and mix it with 1200 µL extraction buffer and 700 µL phenol:chloroform:isoamyl alcohol (25:24:1). Mix the tubes until the slurry is homogeneous and transfer them to a HulaMixer for 10 min at room temperature.
2. Centrifuge the mixtures at 14,000 xg for 5 min at room temperature and transfer the aqueous phase containing the HMW DNA into four new 2 mL Eppendorf tubes.
3. Add 4 µL of RNase A to the solution and mix the tubes carefully. Incubate for 30 min at 50°C.
4. Add equal volumes of phenol:cholroform and mix carefully. Centrifuge at 14,000 xg for 5 min at room temperature.
5. Transfer the aqueous layer to the same 15 mL falcon tube and add equal amounts of binding buffer to the tube.
6. Continue to section “Purification of HMW DNA”.

Extraction of HMW DNA using Genomic Buffer Set from QIAGEN

The following method is from the manufacture’s protocol with small modifications.

1. Transfer 25 mg of lyophilized and ground mycelium into each of three 2 mL Eppendorf tubes and mix them with 1 mL Buffer Y1 containing β-mercaptoethanol, and subsequently add Lysing Enzyme from

Trichoderma harzianum for a final concentration of 5.5 mg/mL. Mix the tubes until the solution is homogeneous and incubate it at 37°C for 1 h.
2. Pellet the degraded cells by centrifugation at 5,000 xg for 10 min at room temperature. Remove the supernatant and carefully resuspend the pellet in 2 mL Buffer G2 containing 4 µL RNase A and 45 µL Proteinase K by mixing the tubes. Incubate the mixtures at 50°C for 2 h.
3. Centrifuge the tubes at 5,000 xg for 10 min at room temperature.
4. Continue to section "Purification of HMW DNA".

Purification of HMW DNA

The following method is from the manufacture's protocol with small modifications.

1. Equilibrate the QIAGEN Genomic-Tips 20/G with 1 mL of Buffer QBT from the Genomic Buffer Set
2. Load 2 mL of the HMW DNA onto the equilibrated QIAGEN Genomic-Tips 20/G.
3. Repeat the step above until all the HMW DNA is loaded onto the QIAGEN Genomic-Tips 20/G.
4. Wash the HMW DNA with 1 mL of Buffer QC from the Genomic Buffer Set.
5. Repeat the step above three times.
6. Elute the HMW DNA in a 1.5 mL Eppendorf tube with 1 mL of Buffer QF from the Genomic Buffer Set.
7. Precipitate the eluted HMW DNA by mixing it with 700 µL isopropanol and carefully mix by turning the tubes upside down. Incubate the mixture for 30 min at room temperature.
8. Centrifuge the precipitated HMW DNA for 15 min at 14,000 xg and carefully remove supernatant without disrupting the pellet.
9. Wash the pellet with 1 mL ice-cold ethanol without disputing it and centrifuge for 5 min at 14,000 xg. Remove the ethanol.
10. Repeat the step above one more time.
11. Be sure to remove all of the ethanol from the pellet and let the pellet dry for 30 sec at room temperature.
12. Dissolve the pellet in 65 µL tris-HCl buffer.
13. Gently flip the tubes and let them mix on a HulaMixer overnight.
14. Continue to section "Quality control of HMW DNA".

Quality control of HMW DNA

1. Measure the DNA concentration, the A260/A230 ratio, and the A260/A280 ratio on NanoDrop One (ThermoFisher) or similar.
2. Measure the DNA concentration (e.g. using Qubit 3.0 (Invitrogen) with Qubit™ dsDNA HS Assay Kit or similar)
3. Use the TapeStation 2200 (Agilent) with the Genomic DNA ScreenTape or similar to determine the length of the fragments and the DNA Integrity Number (DIN).

Removal of small fragments using Circulomics Short Read Eliminator XS

The following method is from the manufacture's protocol with minute modifications. In our hands, it has proven useful to include a size select step even if the DIN measure is above 8 to get highest possible N50 of the read length.

1. Transfer 1.5 - 9 µg DNA to a 1.5 mL Eppendorf tube and adjust the volume to 60 µL in tris-HCl buffer and add 60 µL of Buffer SRE XS to the tube. Carefully mix by flicking the tubes and incubate for 30 min at room temperature.
2. Centrifuge the mixture at 10,000 xg for 30 min and remove the liquid. Be careful not to disturb the pellet.

3. Wash the pellet with 80 % ethanol without disrupting it and centrifuge it at 10,000 xg for 2 min. Remove the ethanol.
4. Repeat the step above. Be sure to remove all of the liquid from the pellet and let the pellet dry for 1 min at room temperature.
5. Dissolve the HMW DNA in 60 µL EB buffer.
6. Gently flip the tubes and let them mix on a HulaMixer overnight to allow complete dissolution.
7. Continue to section "Quality control of HMW DNA".

Sequencing

The amount of HMW DNA required depends on the flow cell version, whether the samples are multiplexed, and the fragment distribution. We recommend a fragment distribution with a mean of >60 kbp and a DIN >8, a A260/A230 ratio of 1.8 and a A260/A280 ratio between 2.0 – 2.2 and a ratio of concentration estimates from NanoDrop and Qubit between 1 – 1.5. Use the protocol "Genomic DNA by Ligation (SQK-LSK109)" from ONT (Oxford, UK) if sequencing one fungal genome. Use the protocol "Native barcoding genomic DNA (with EXP-NBD104, EXPNBD114, and SQK-LSK109)" from ONT (Oxford, UK) if several fungal genomes will be sequenced on the same flow cell.

Notes

1. The composition of the extraction buffer is rather simple in comparison to some buffers that has been used in DNA extraction procedures from fungi. It is indeed possible that more complex extraction buffers will result in a higher yield, at least for some fungi. Since this buffer provides acceptable yield which is sufficient for nanopore sequencing, we prioritized simplicity.

Supplementary note 3

Table S1: Overview of concentration, purity, fragment length and DIN of the extracted DNA used for sequencing after size selection using Circulomics Short Read Eliminator XS.

	Apiospora pterospermum	*Aspergillus westerdijkiae*	*Penicillium aurantiogriseum*	*Aspergillus sp. (subgen. Cremei)*
DNA extraction method	Genomic Buffer Set	Genomic Buffer Set	Genomic Buffer Set	Phenol-chloroform
Purification method	Genomic-Tips 20/G	Genomic-Tips 20/G	Genomic-Tips 20/G	Genomic-Tips 20/G
DNA conc. (Qubit) (ng/µL)	206	68.8	125	71.0
DNA conc. (Nanodrop) (ng/µL)	377	104	184	86
DNA concentration (Qubit) (ng/µL)	206	68.8	125	71.0
A260/230 ratio	1.9	1.9	1.9	1.9
A260/280 ratio	2.3	2.3	2.3	2.4
NanoDrop/Qubit ratio	1.8	1.5	1.5	1.2
Fragment length (bp)	>60000	>60000	>60000	59418
DIN	9.5	9.4	9.8	8.9

Supplementary note 4

Table S2: Repetitive elements and RNA genes in Apiospora pterospermum with 125x coverage.

Repeat type		Number of elements	Length occupied (b)	Percentages of genome (%)
Interspersed repeat	SINEs	1397	71078	0.16
	LINEs	3410	212979	0.48
	LTR elements	16362	1184757	2.66
	DNA elements	10207	592544	1.33
Tandem Repeat		11368	777399	1.75
Type	Number	Average length (b)	Total length (b)	Percentages in genome (%)
tRNA	203	92	18696	0.052
28s rRNA	6	4875	29251	0.081
8s rRNA	6	152	912	0.003
18s RNA	6	1800	10800	0.030

Table S3: Repetitive elements and RNA genes in Aspergillus westerdijkiae with 130x coverage.

Repeat type		Number of elements	Length occupied (b)	Percentages of genome (%)
Interspersed repeat	SINEs	1265	62658	0.19
	LINEs	3884	270288	0.84
	LTR elements	11965	886626	2.76
	DNA elements	10721	699407	2.17
Tandem Repeat		5161	660066	1.83
Type	Number	Average length (b)	Total length (b)	Percentages in genome (%)
tRNA	230	86	19746	0.055
28s rRNA	7	3686	25801	0.072
8s rRNA	7	152	1063	0.003
18s RNA	7	1798	12583	0.035

Table S4: Repetitive elements and RNA genes in Penicillium aurantiogriseum with 139x coverage.

Repeat type		Number of elements	Length occupied (bp)	Percentages of genome (%)
Interspersed repeat	SINEs	1162	61383	0.19
	LINEs	3145	221590	0.68
	LTR elements	10084	748791	2.29
	DNA elements	9913	649404	1.99
Tandem Repeat		8579	719958	2.21
Type	Number	Average length (bp)	Total length	Percentages in genome (%)
tRNA	210	89	18622	0.052
28s rRNA	5	3675	18376	0.051
8s rRNA	5	151	754	0.002
18s RNA	5	1797	8986	0.025

Table S5: Repetitive elements and RNA genes in Aspergillus sp. (subgen. Cremei) with 91x coverage.

Repeat type		Number of elements	Length occupied (bp)	Percentages of genome (%)
Interspersed repeat	SINEs	1443	72295	0.20
	LINEs	3794	248472	0.69
	LTR elements	14127	989179	2.74
	DNA elements	11356	668504	1.85
Tandem Repeat		5161	332720	1.03
Type	Number	Average length (bp)	Total length	Percentages in genome (%)
tRNA	236	84	19784	0.055
28s rRNA	5	3613	18065	0.050
8s rRNA	4	152	607	0.002
18s RNA	4	1798	7192	0.020

Supplementary note 5

Table S6: Overview of BUSCO analysis of polishing steps of all assemblies with different coverage. See materials and methods for details. C (%), S (%), D (%), F (%), and M (%) denotes BUSCO completeness in percent, complete and single-copy BUSCOs in percent, complete and duplicated BUSCOs in percent, fragmented BUSCOs in percent, and missing BUSCOs in percent, respectively.

Species	Assembly	Coverage (x)	C (%)	S (%)	D (%)	F (%)	M (%)
P. aurantiogriseum	Racon+Medakax2	139	97.5	97.3	0.2	1.1	1.4
P. aurantiogriseum	Racon+Medaka	139	97.5	97.2	0.3	0.8	1.7
P. aurantiogriseum	Racon	139	90.8	90.6	0.2	4.9	4.3
P. aurantiogriseum	None	139	1.3	1.3	0	9.4	89.3
P. aurantiogriseum	Racon+Medakax2	101	97.9	97.5	0.4	0.9	1.2
P. aurantiogriseum	Racon+Medaka	101	97.3	96.9	0.4	1.1	1.6
P. aurantiogriseum	Racon	101	90.7	90.4	0.3	5.8	3.5
P. aurantiogriseum	None	101	1.4	1.4	0	9	89.6
P. aurantiogriseum	Racon+Medakax2	76	97.6	97.3	0.3	1	1.4
P. aurantiogriseum	Racon+Medaka	76	97.5	97.2	0.3	1	1.5
P. aurantiogriseum	Racon	76	90.4	90.2	0.2	5.3	4.3
P. aurantiogriseum	None	76	1.1	1.1	0	7.8	91.1
P. aurantiogriseum	Racon+Medakax2	50	97	96.7	0.3	1.1	1.9
P. aurantiogriseum	Racon+Medaka	50	96.7	96.4	0.3	1.3	2.0
P. aurantiogriseum	Racon	50	91.5	91.3	0.2	4.1	4.4
P. aurantiogriseum	None	50	1	1	0	9.2	89.8
P. aurantiogriseum	Racon+Medakax2	25	95.2	95	0.2	2.4	2.4
P. aurantiogriseum	Racon+Medaka	25	95	94.8	0.2	2.6	2.4
P. aurantiogriseum	Racon	25	89.4	89.2	0.2	4.7	5.9
P. aurantiogriseum	None	25	0.8	0.8	0	8.3	90.9
P. aurantiogriseum	Racon+Medakax2	12	72.8	72.6	0.2	7.6	19.6
P. aurantiogriseum	Racon+Medaka	12	71.8	71.6	0.2	8.1	20.1
P. aurantiogriseum	Racon	12	66.1	65.9	0.2	11.9	22
P. aurantiogriseum	None	12	1.4	1.4	0	6.5	92.1
A. pterospermum	Racon+Medakax2	125	98	97.9	0.1	0.8	1.2
A. pterospermum	Racon+Medaka	125	98	97.9	0.1	0.8	1.2
A. pterospermum	Racon	125	90	90	0	5.5	4.5
A. pterospermum	None	125	2.2	2.2	0	7.2	90.6
A. pterospermum	Racon+Medakax2	101	97.8	97.7	0.1	0.7	1.5
A. pterospermum	Racon+Medaka	101	97.7	97.6	0.1	0.9	1.4
A. pterospermum	Racon	101	90.7	90.5	0.2	5.6	3.7
A. pterospermum	None	101	2.4	2.4	0	7.1	90.5
A. pterospermum	Racon+Medakax2	75	97.7	97.6	0.1	0.8	1.5
A. pterospermum	Racon+Medaka	75	97.3	97.2	0.1	1.2	1.5
A. pterospermum	Racon	75	90.1	90.1	0	4.8	5.1
A. pterospermum	None	75	3	3	0	8.3	88.7

A. pterospermum	Racon+Medakax2	51	97.5	97.4	0.1	1.1	1.4
A. pterospermum	Racon+Medaka	51	97.2	97.1	0.1	1.1	1.7
A. pterospermum	Racon	51	89.9	89.8	0.1	5.6	4.5
A. pterospermum	None	51	2.1	2.1	0	9.7	88.2
A. pterospermum	Racon+Medakax2	25	95.8	95.6	0.2	2.1	2.1
A. pterospermum	Racon+Medaka	25	95.7	95.5	0.2	1.8	2.5
A. pterospermum	Racon	25	88	87.8	0.2	6.0	6.0
A. pterospermum	None	25	2.9	2.9	0	7.5	89.6
A. pterospermum	Racon+Medakax2	12	76.5	76.3	0.2	7.2	16.3
A. pterospermum	Racon+Medaka	12	76.2	76	0.2	7.1	16.7
A. pterospermum	Racon	12	65.8	65.6	0.2	13.4	20.8
A. pterospermum	None	12	2.7	2.7	0.0	10.7	86.6
A. westerdijkiae	Racon+Medakax2	130	97.9	97.4	0.5	0.7	1.4
A. westerdijkiae	Racon+Medaka	130	98	97.5	0.5	0.7	1.3
A. westerdijkiae	Racon	130	88.3	88	0.3	5.8	5.9
A. westerdijkiae	None	130	1.6	1.6	0	8.3	90.1
A. westerdijkiae	Racon+Medakax2	100	97.3	96.8	0.5	1.1	1.6
A. westerdijkiae	Racon+Medaka	100	97.8	97.4	0.4	0.7	1.5
A. westerdijkiae	Racon	100	87.8	87.5	0.3	6.2	6
A. westerdijkiae	None	100	1.8	1.8	0	11	87.2
A. westerdijkiae	Racon+Medakax2	75	97.7	97.2	0.5	1	1.3
A. westerdijkiae	Racon+Medaka	75	97.3	96.7	0.6	1.3	1.4
A. westerdijkiae	Racon	75	88.3	88.1	0.2	5.9	5.8
A. westerdijkiae	None	75	1.4	1.4	0	11.3	87.3
A. westerdijkiae	Racon+Medakax2	50	96.7	96.3	0.4	1.1	2.2
A. westerdijkiae	Racon+Medaka	50	97	96.7	0.3	1	2
A. westerdijkiae	Racon	50	87.5	87.3	0.2	6.2	6.3
A. westerdijkiae	None	50	1.7	1.7	0	10	88.3
A. westerdijkiae	Racon+Medakax2	25	94.6	94.1	0.5	1.9	3.5
A. westerdijkiae	Racon+Medaka	25	94	93.5	0.5	2.8	3.2
A. westerdijkiae	Racon	25	84.2	84	0.2	7.6	8.2
A. westerdijkiae	None	25	1.7	1.7	0	11	87.3
A. westerdijkiae	Racon+Medakax2	11	77.2	76.8	0.4	7.7	15.1
A. westerdijkiae	Racon+Medaka	11	75.7	75.3	0.4	8.6	15.7
A. westerdijkiae	Racon	11	66.1	65.9	0.2	13.3	20.6
A. westerdijkiae	None	11	1.2	1.2	0	9.7	89.1
A. cf. subgen. Cremei	Racon+Medakax2	91	97.8	97.4	0.4	0.5	1.7
A. cf. subgen. Cremei	Racon+Medaka	91	97.6	97.2	0.4	0.5	1.9
A. cf. subgen. Cremei	Racon	91	87.1	86.8	0.3	6.9	6
A. cf. subgen. Cremei	None	91	1.1	1.1	0	7.6	91.3
A. cf. subgen. Cremei	Racon+Medakax2	75	97.4	97	0.4	0.8	1.8

A. cf. subgen. Cremei	Racon+Medaka		75	97.9	97.5	0.4	0.6	1.5
A. cf. subgen. Cremei	Racon		75	88.1	87.8	0.3	6.1	5.8
A. cf. subgen. Cremei	None		75	1.4	1.4	0	8.2	90.4
A. cf. subgen. Cremei	Racon+Medakax2		50	97.4	97	0.4	0.9	1.7
A. cf. subgen. Cremei	Racon+Medaka		50	97.3	96.9	0.4	1.1	1.6
A. cf. subgen. Cremei	Racon		50	87.5	87.2	0.3	5.7	6.8
A. cf. subgen. Cremei	None		50	1.1	1.1	0	7.1	91.8
A. cf. subgen. Cremei	Racon+Medakax2		25	95.2	94.8	0.4	2	2.8
A. cf. subgen. Cremei	Racon+Medaka		25	95.1	94.8	0.3	2.1	2.8
A. cf. subgen. Cremei	Racon		25	84.4	84.3	0.1	7.8	7.8
A. cf. subgen. Cremei	None		25	1.4	1.4	0	8.2	90.4
A. cf. subgen. Cremei	Racon+Medakax2		11	71.3	71	0.3	8.4	20.3
A. cf. subgen. Cremei	Racon+Medaka		11	70.6	70.3	0.3	8.5	20.9
A. cf. subgen. Cremei	Racon		11	59.3	59	0.3	13.8	26.9
A. cf. subgen. Cremei	None		11	1.4	1.4	0	6	92.6

Supplementary note 6

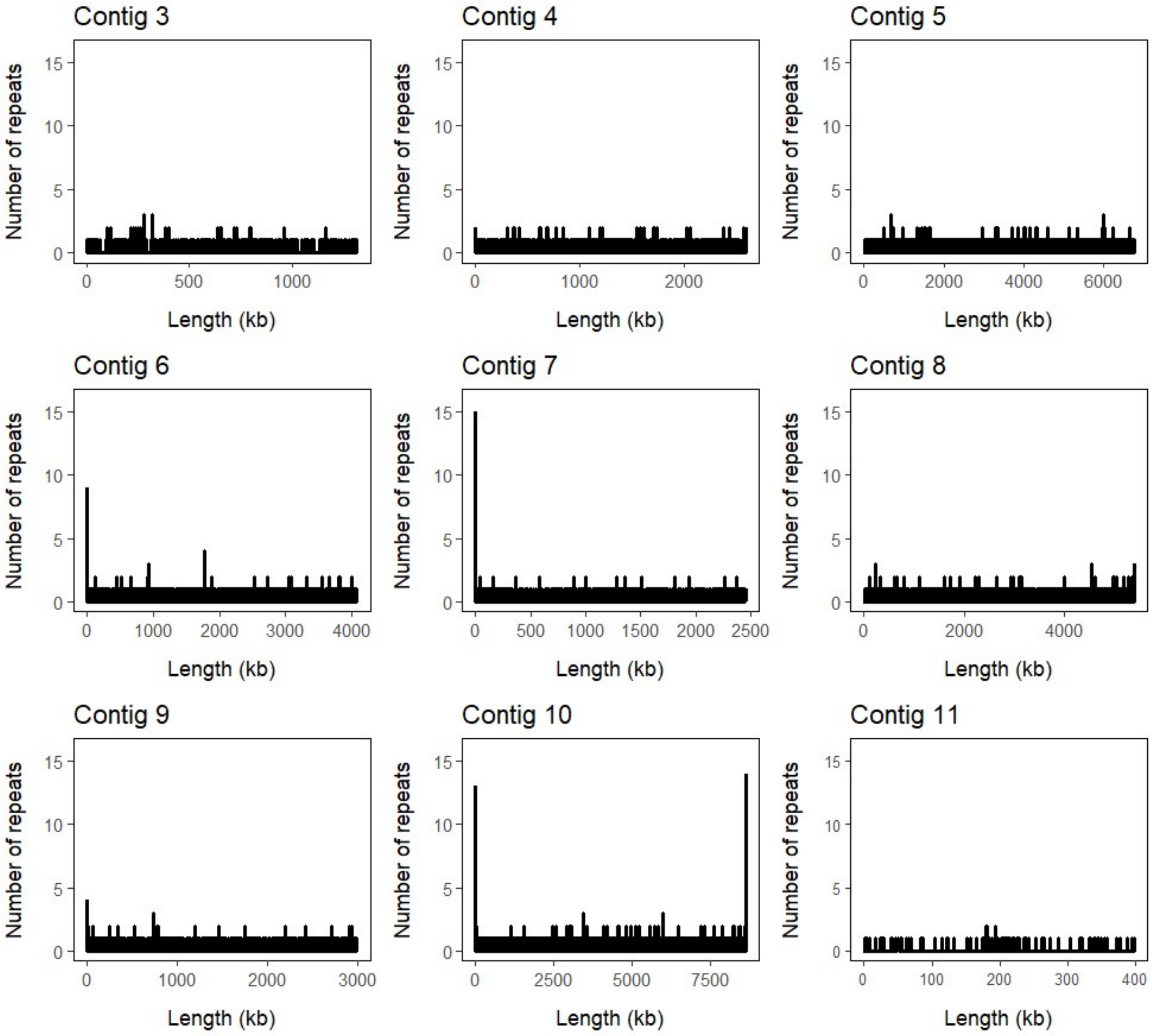

Figure S1: Overview of telomeric regions of contig 3 – 11 in slidning window of 100 b with 25 b increments. These contigs are from the draft made for Apiospora pterospermum with 125x coverage. Contigs comprising mtDNA or exclusively rRNA genes are not included in the analysis.

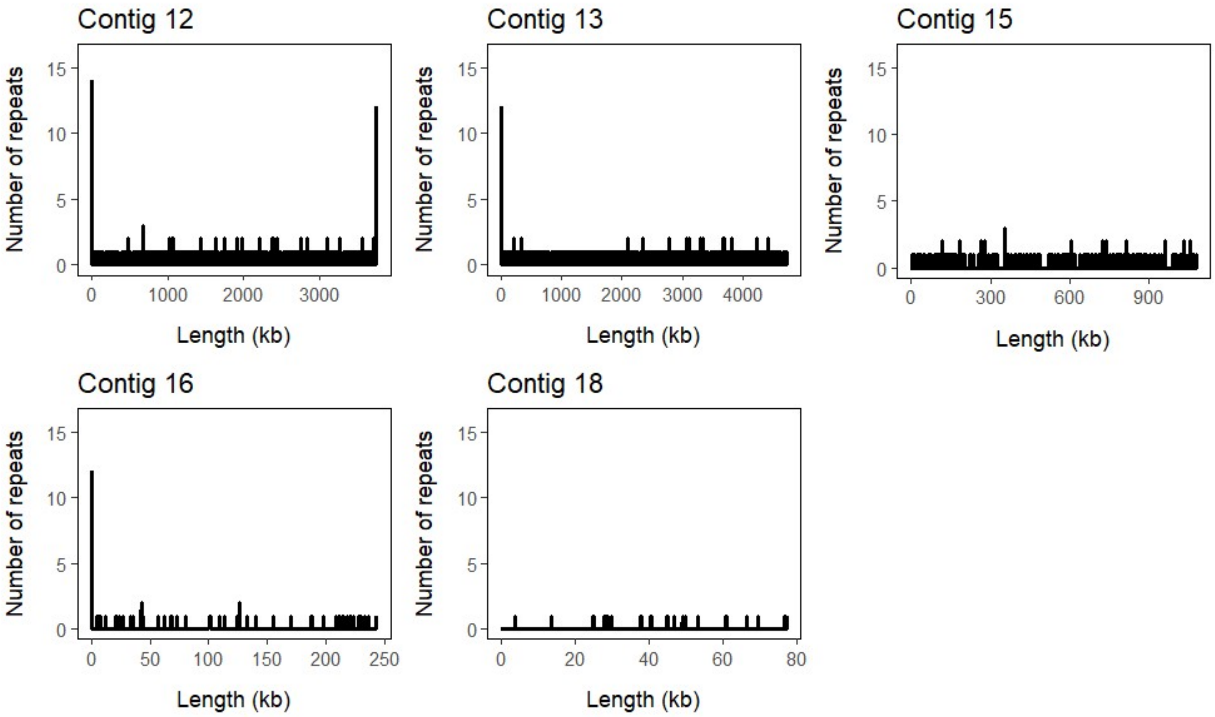

Figure S2: Overview of telomeric regions of contig 12 – 18 in slidning window of 100 b with 25 b increments. These contigs are from the draft made for Apiospora pterospermum with 125x coverage. Contigs comprising mtDNA or exclusively rRNA genes are not included in the analysis.

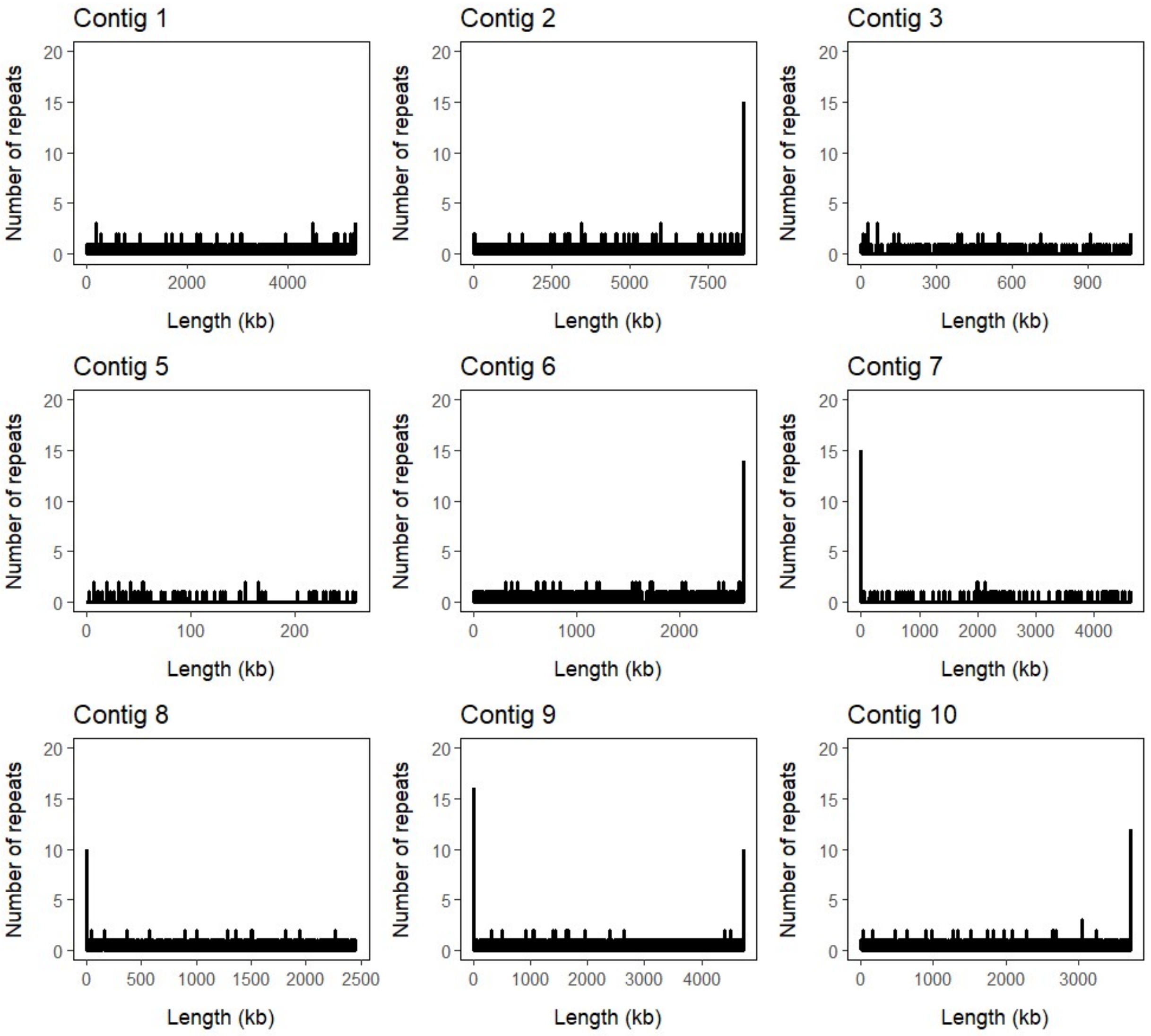

Figure S3: Overview of telomeric regions of contig 1 – 10 in slidning window of 100 b with 25 b increments. These contigs are from the draft made for Apiospora pterospermum with 100x coverage. Contigs comprising mtDNA or exclusively rRNA genes are not included in the analysis.

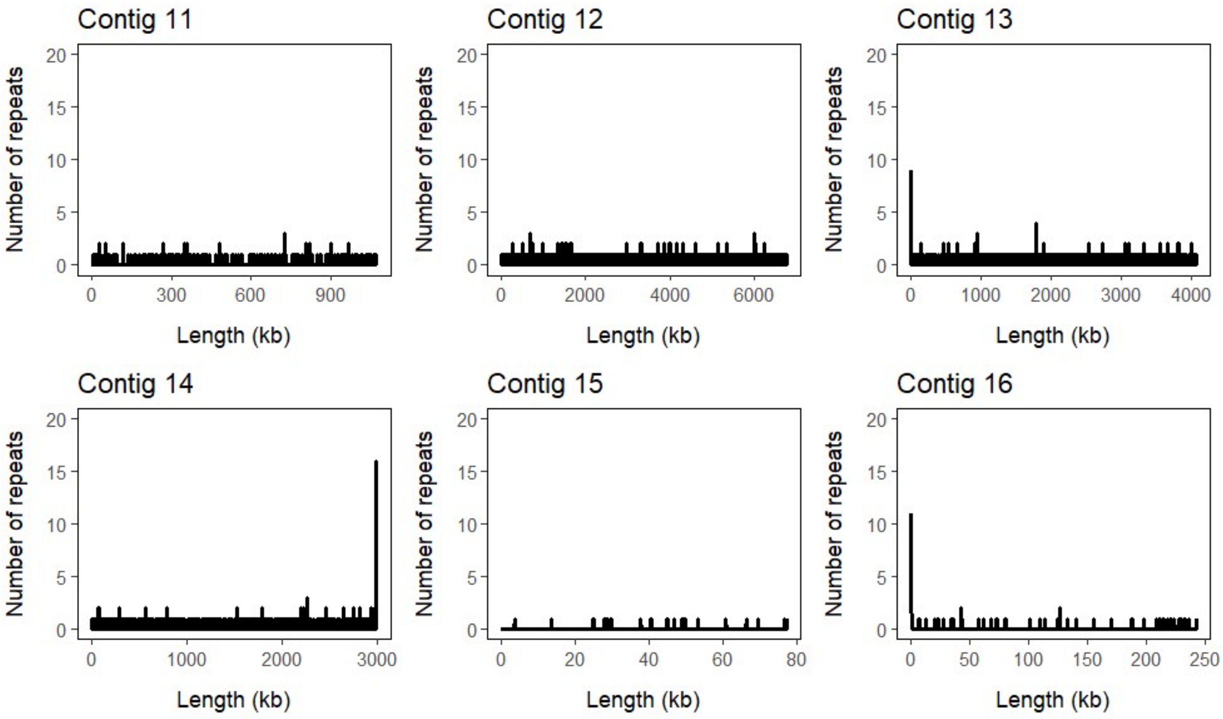

Figure S4: Overview of telomeric regions of contig 11 – 16 in slidning window of 100 b with 25 b increments. These contigs are from the draft made for Apiospora pterospermum with 100x coverage. Contigs comprising mtDNA or exclusively rRNA genes are not included in the analysis.

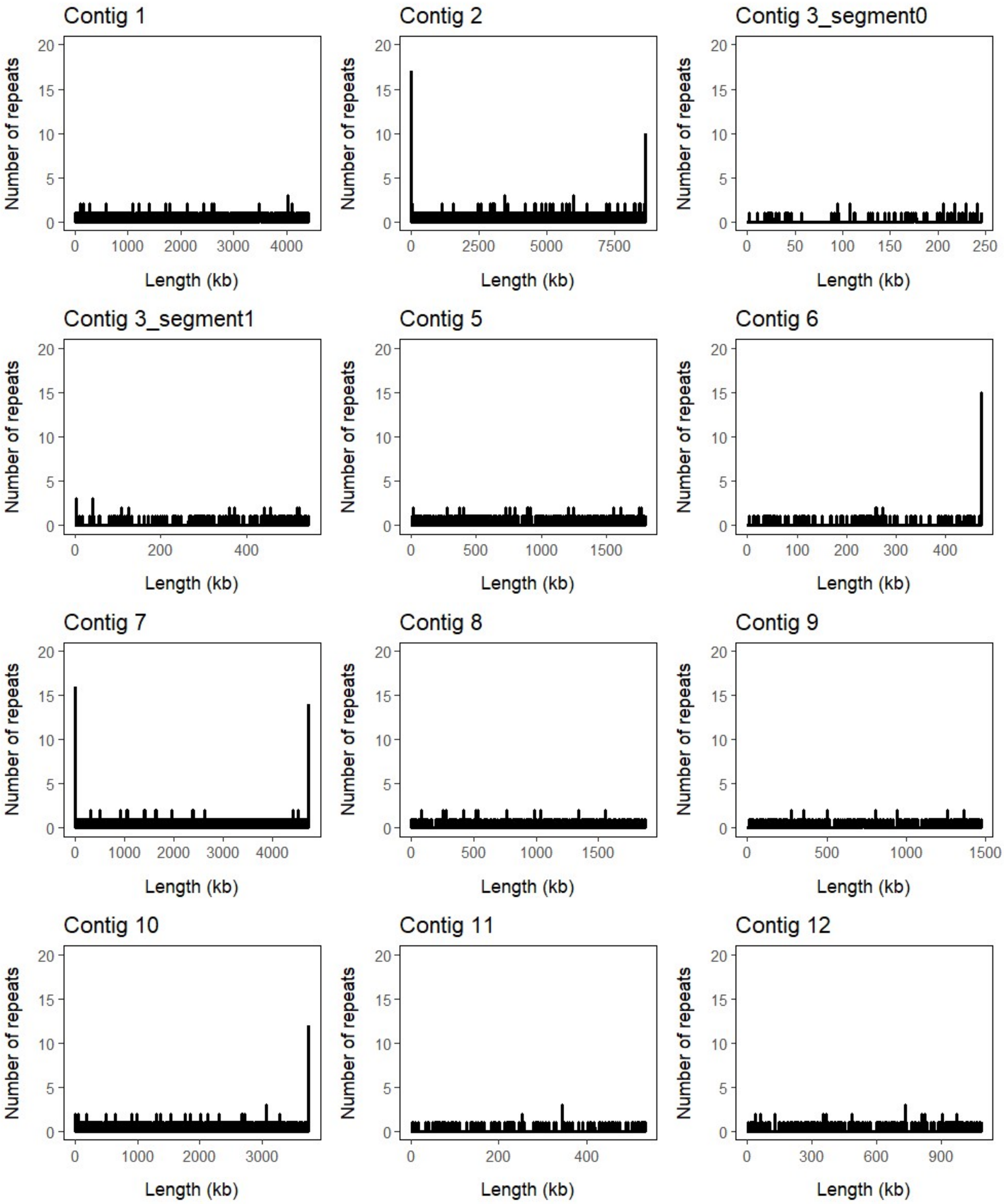

Figure S5: Overview of telomeric regions of contig 1 – 12 in slidning window of 100 b with 25 b increments. These contigs are from the draft made for Apiospora pterospermum with 75x coverage. Contigs comprising mtDNA or exclusively rRNA genes are not included in the analysis. Contig 3 was spilted up in polishing to two new contigs: contig 3_segment0 and contig 3_segment1.

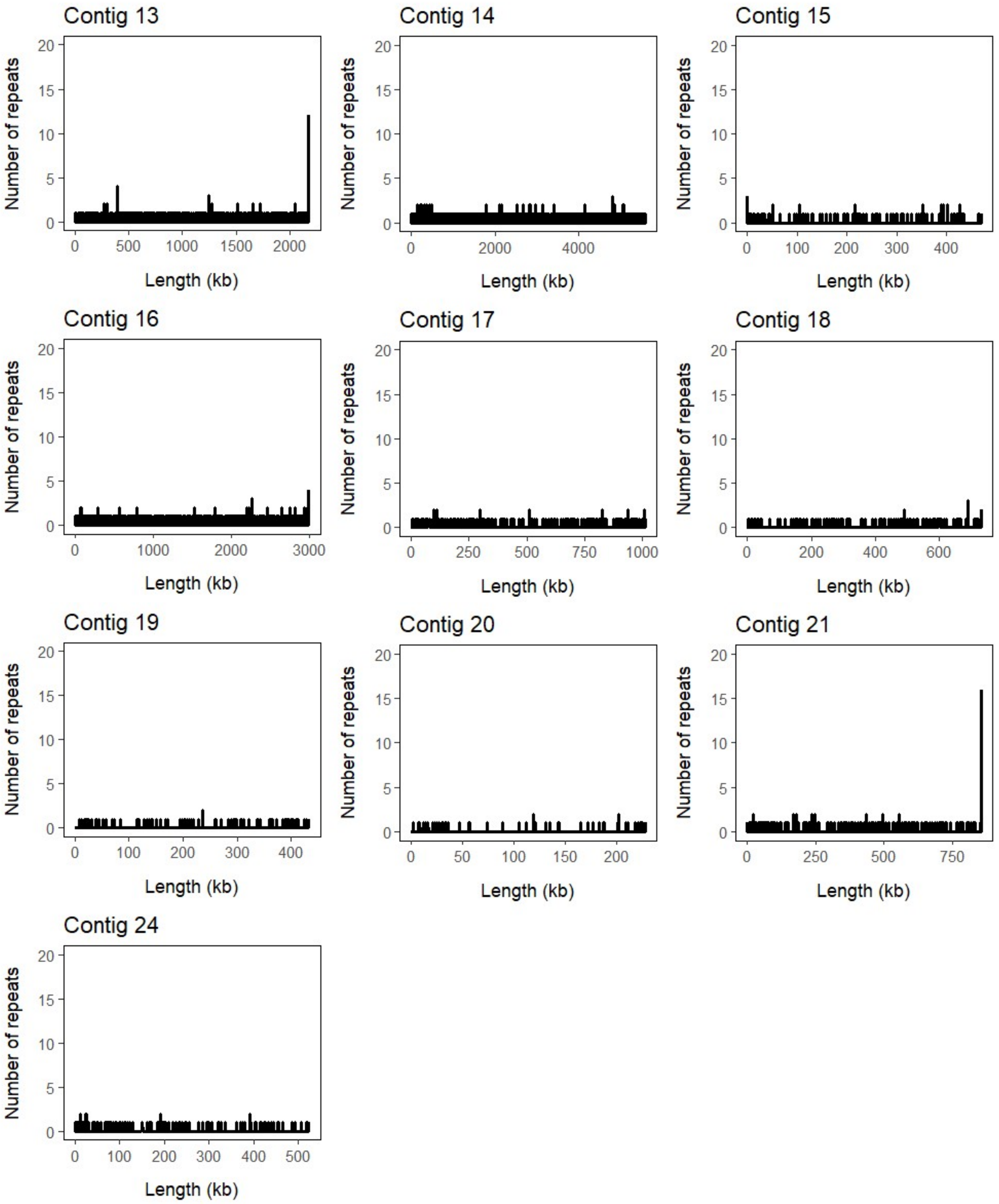

Figure S6: Overview of telomeric regions of contig 13 – 24 in slidning window of 100 b with 25 b increments. These contigs are from the draft made for Apiospora pterospermum with 75x coverage. Contigs comprising mtDNA or exclusively rRNA genes are not included in the analysis.

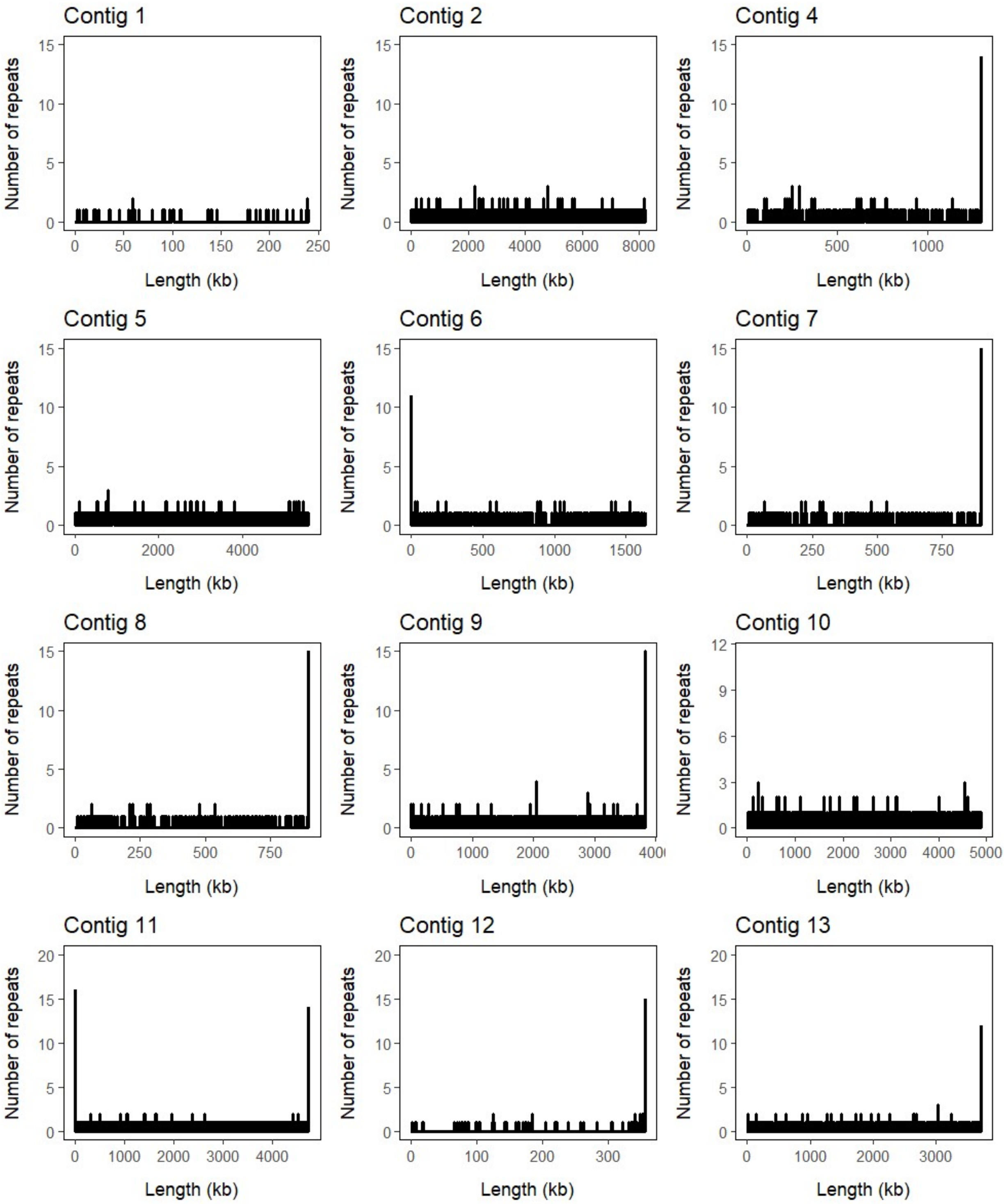

Figure S7: Overview of telomeric regions of contig 1 – 13 in slidning window of 100 b with 25 b increments. These contigs are from the draft made for Apiospora pterospermum with 50x coverage. Contigs comprising mtDNA or exclusively rRNA genes are not included in the analysis.

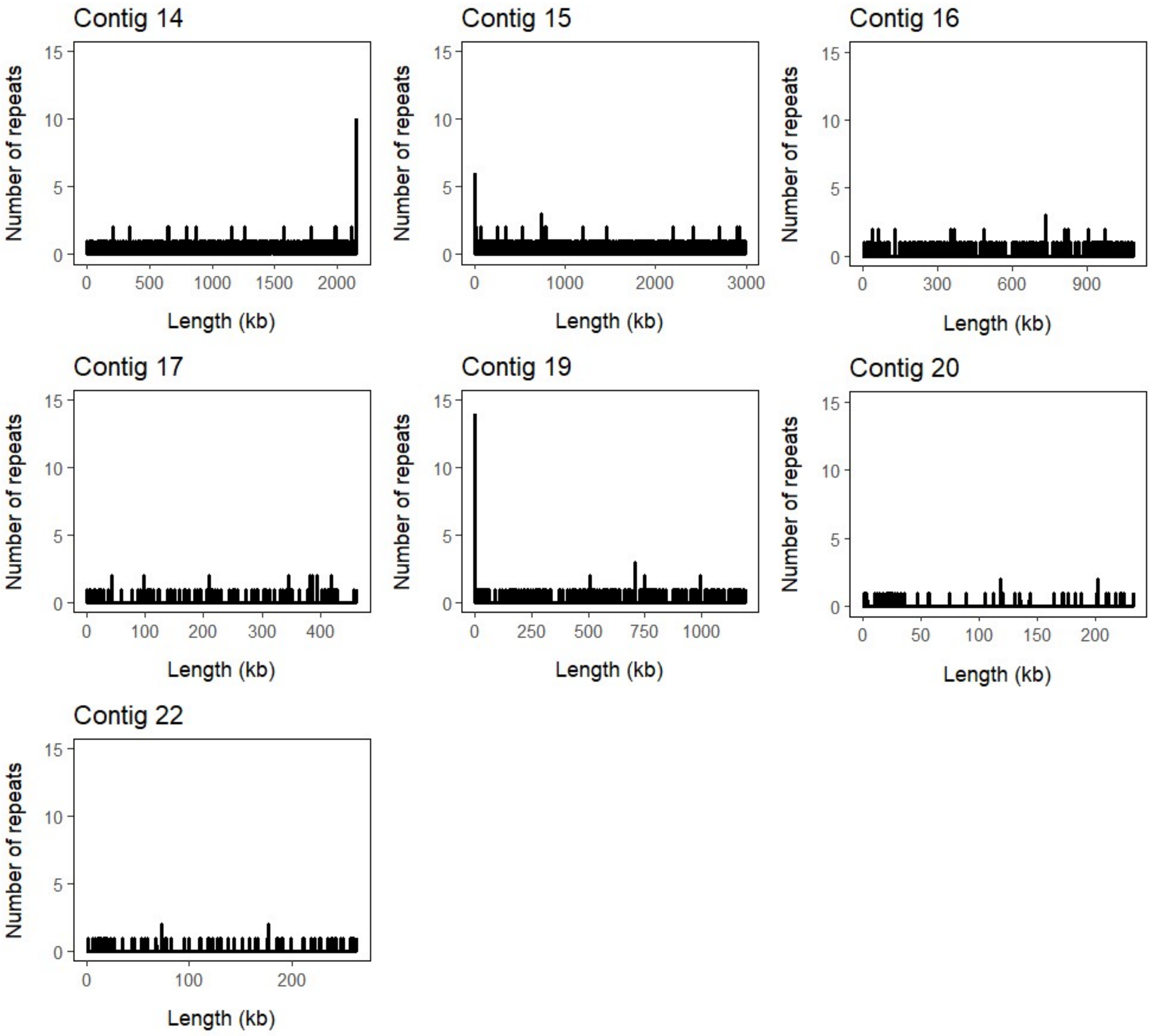

Figure S8: Overview of telomeric regions of contig 14 - 22 in sliding window of 100 b with 25 b increments. These contigs are from the draft made for Apiospora pterospermum with 50x coverage. Contigs comprising mtDNA or exclusively rRNA genes are not included in the analysis.

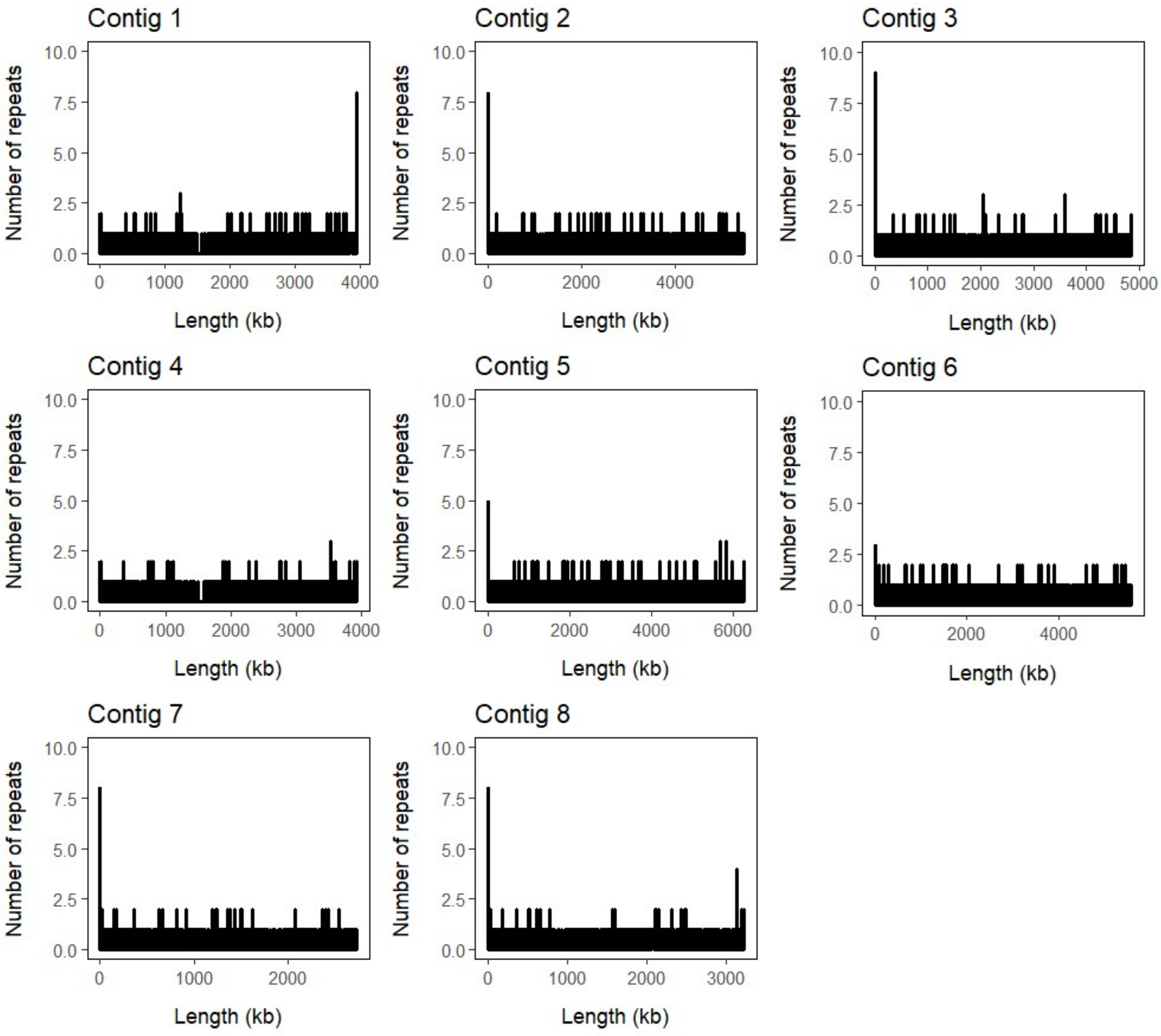

Figure S9: Overview of telomeric regions of a contig in slidning window of 100 b with 25 b increments. These contigs are from the draft made for Aspergillus westerdijkiae with 130x coverage. Contigs comprising mtDNA or exclusively rRNA genes are not included in the analysis.

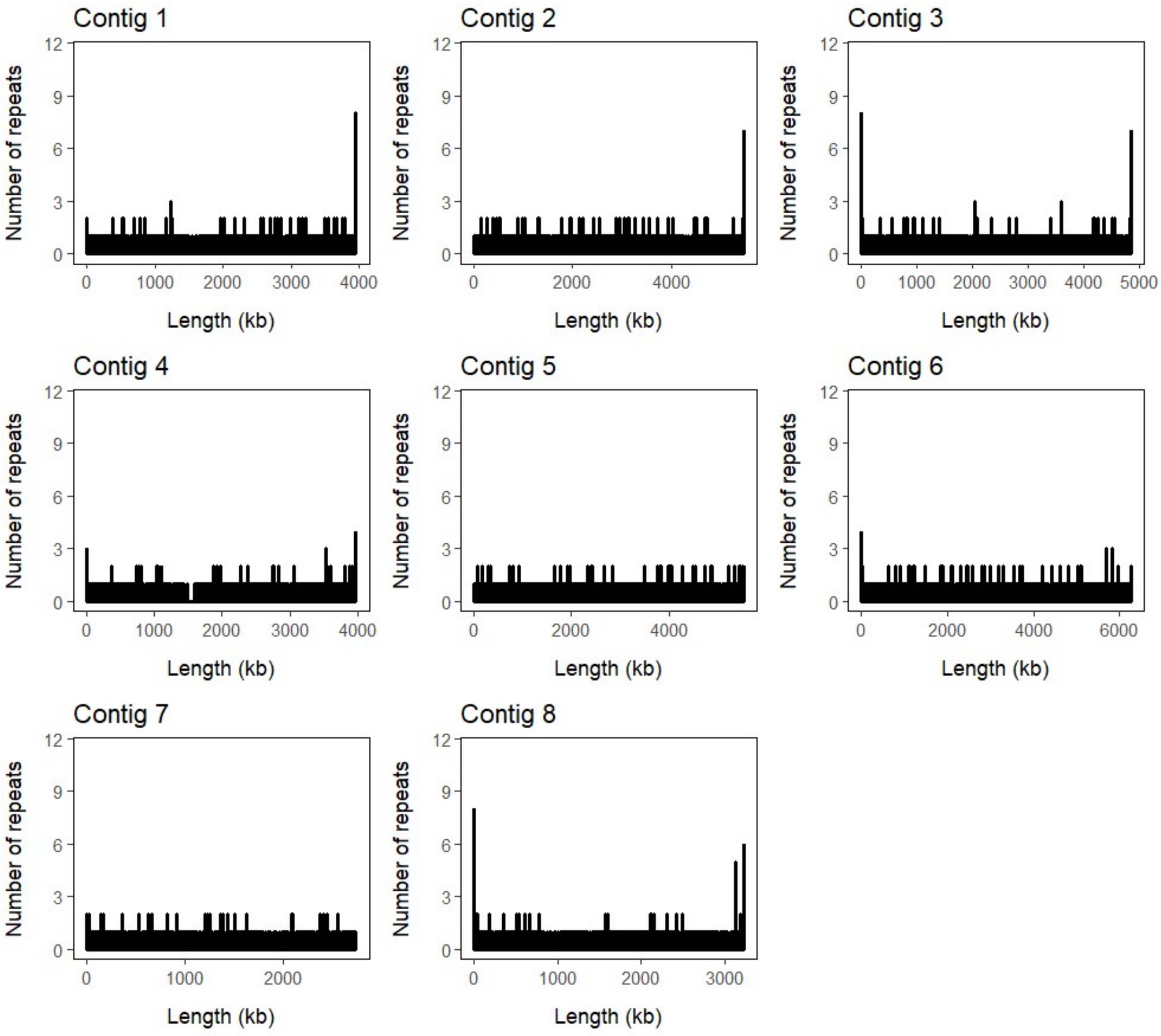

Figure S10: Overview of telomeric regions of a contig in slidning window of 100 b with 25 b increments. These contigs are from the draft made for Aspergillus westerdijkiae with 100x coverage. Contigs comprising mtDNA or exclusively rRNA genes are not included in the analysis.

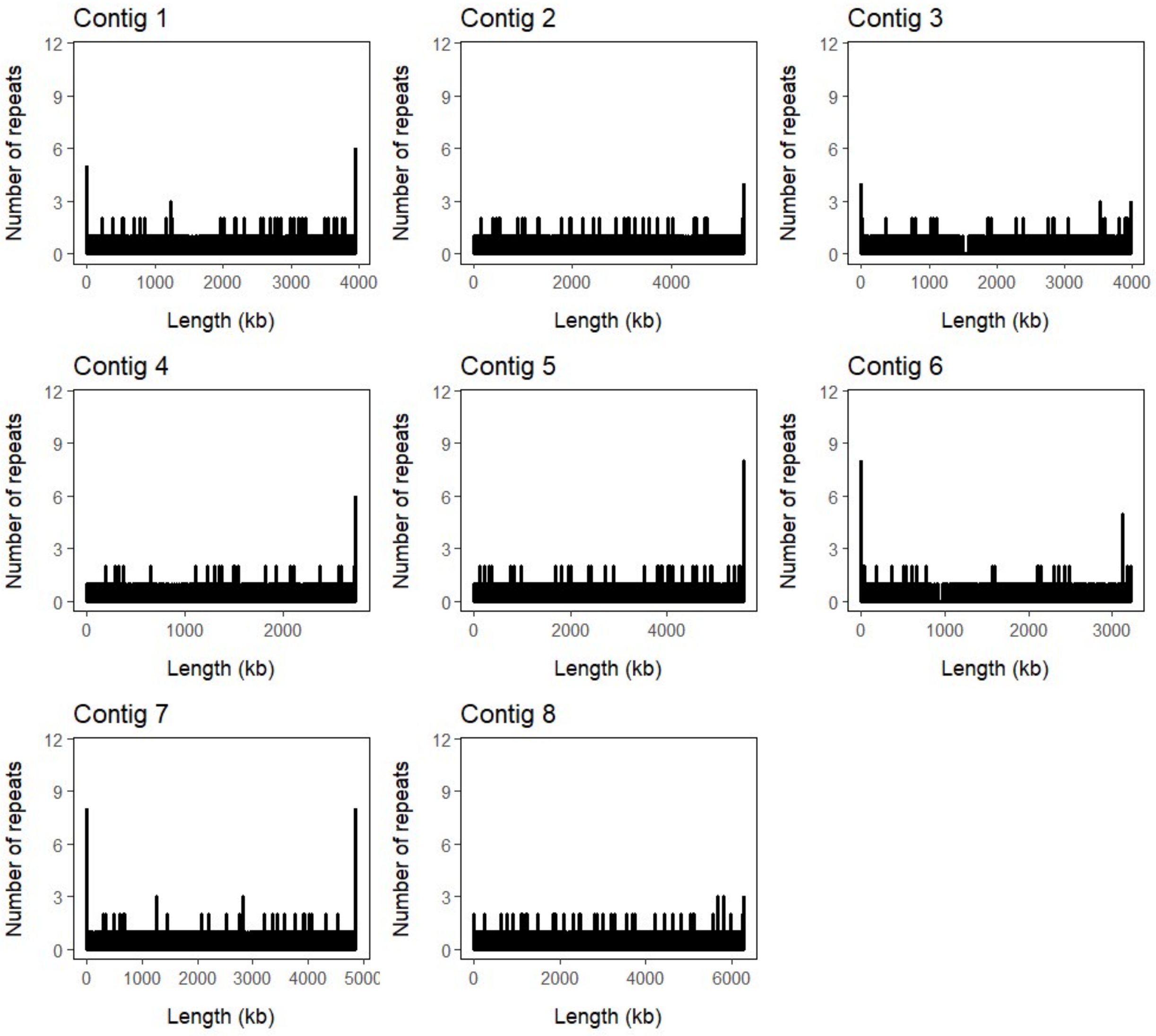

Figure S11: Overview of telomeric regions of a contig in slidning window of 100 b with 25 b increments. These contigs are from the draft made for Aspergillus westerdijkiae with 75x coverage. The increase of number of repeats in the end of contig 6 is not continuous and is therefore not interpreted as a telomeric region. Contigs comprising mtDNA or exclusively rRNA genes are not included in the analysis.

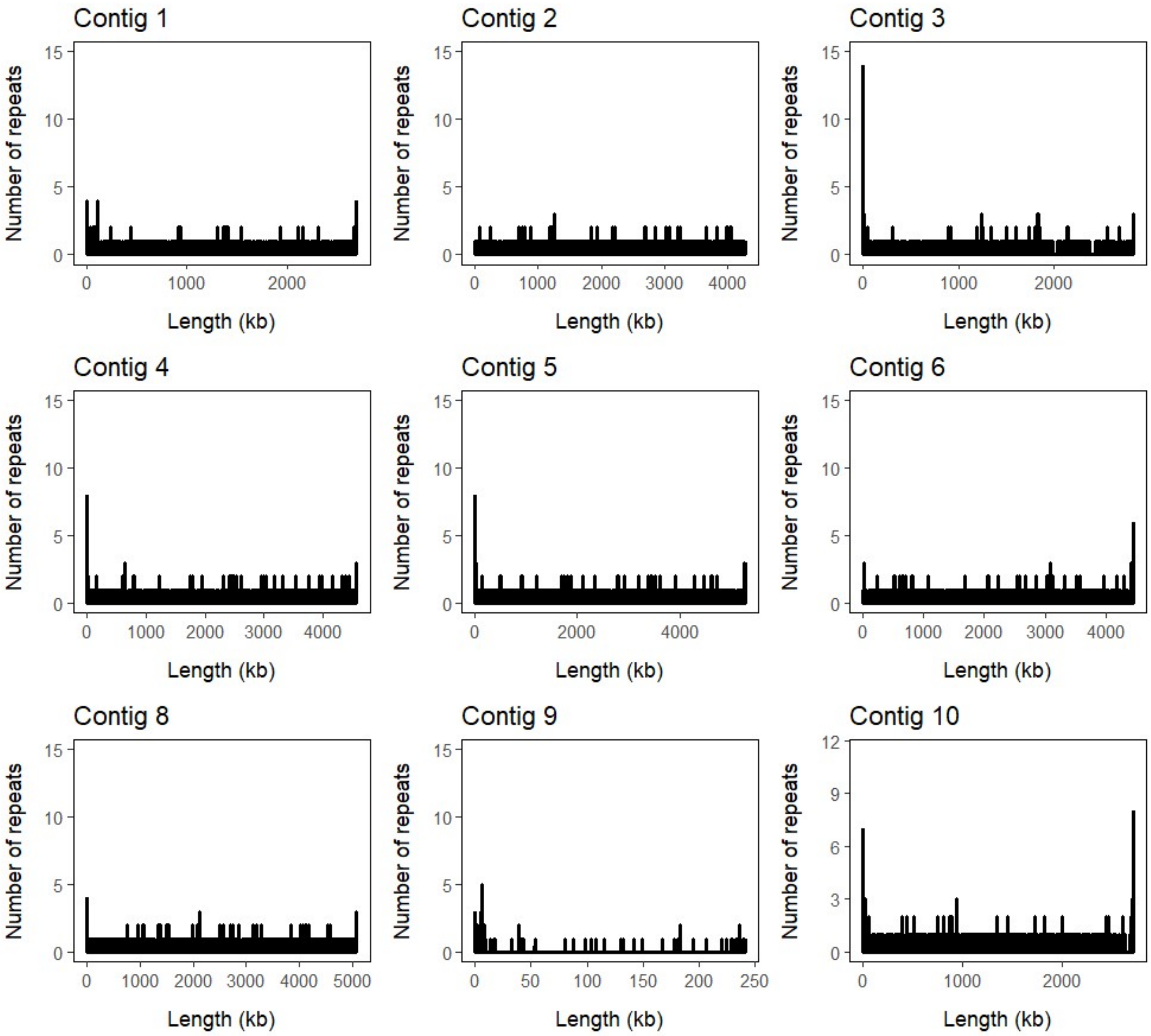

Figure S12: Overview of telomeric regions of a contig in slidning window of 100 b with 25 b increments. These contigs are from the draft made for Aspergillus westerdijkiae with 50x coverage. Contigs comprising mtDNA or exclusively rRNA genes are not included in the analysis.

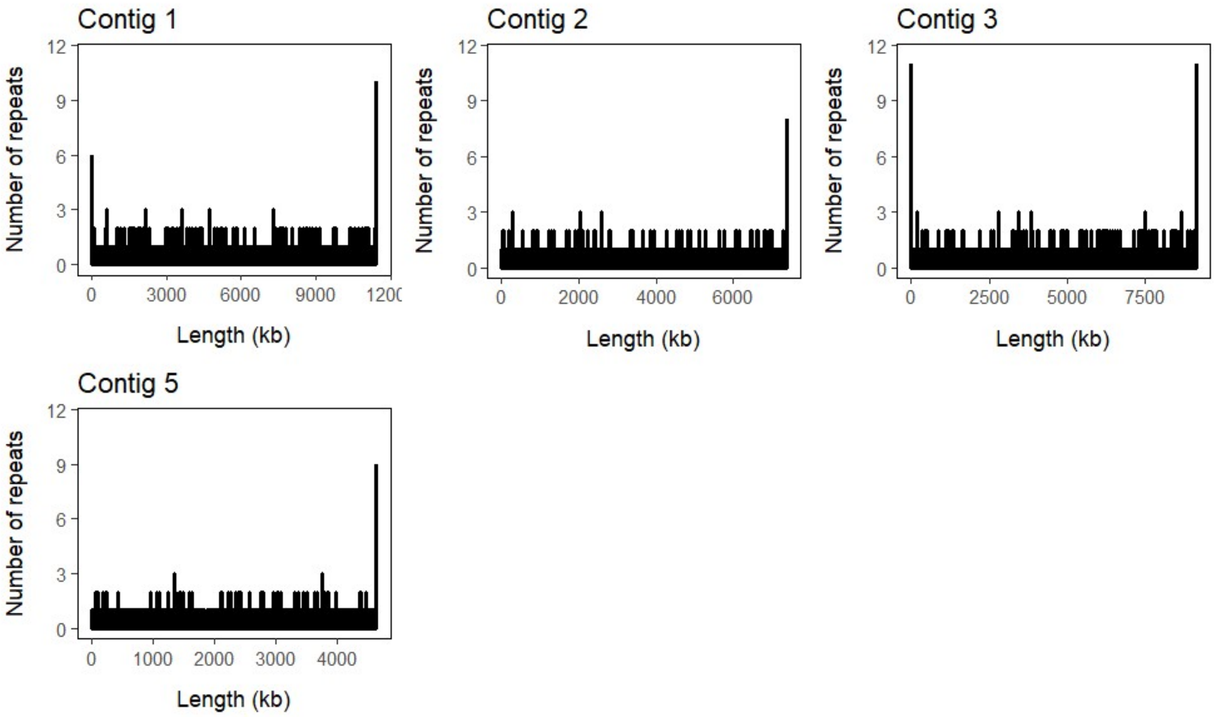

Figure S13: Overview of telomeric regions of a contig in slidning window of 100 b with 25 b increments. These contigs are from the draft made for Penicillium aurantiogriseum with 139x coverage. Contigs comprising mtDNA or exclusively rRNA genes are not included in the analysis.

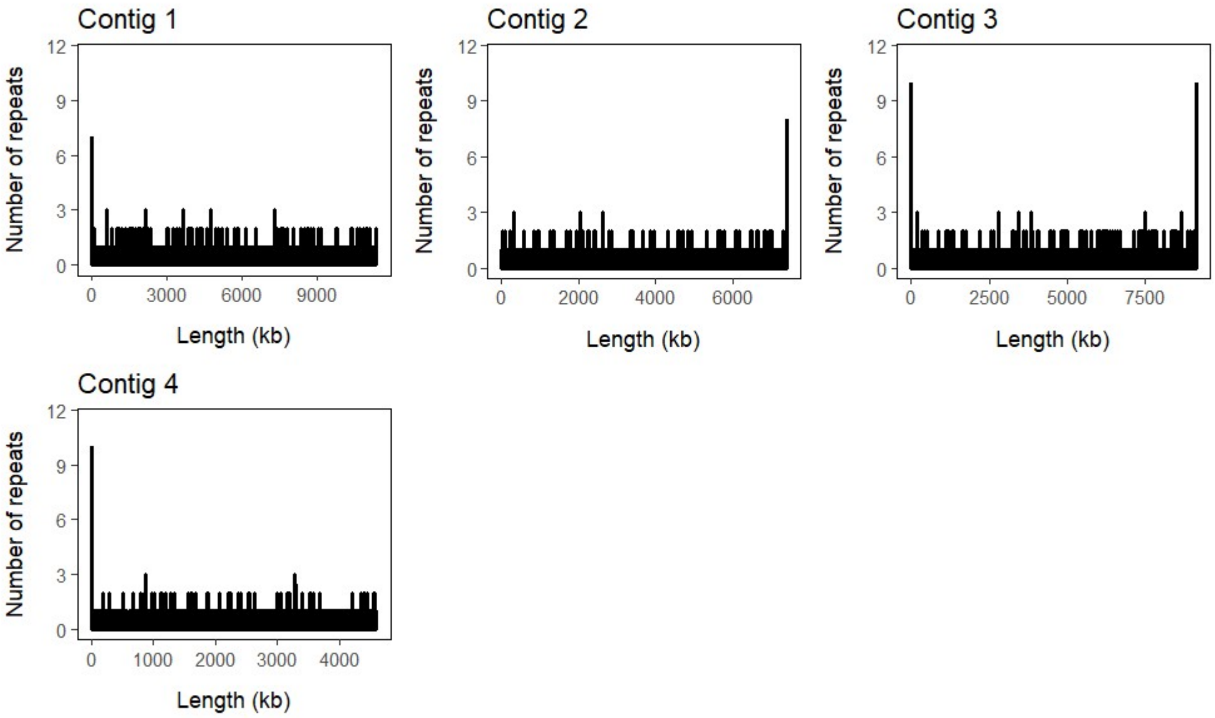

Figure S14: Overview of telomeric regions of a contig in slidning window of 100 b with 25 b increments. These contigs are from the draft made for Penicillium aurantiogriseum with 100x coverage. Contigs comprising mtDNA or exclusively rRNA genes are not included in the analysis.

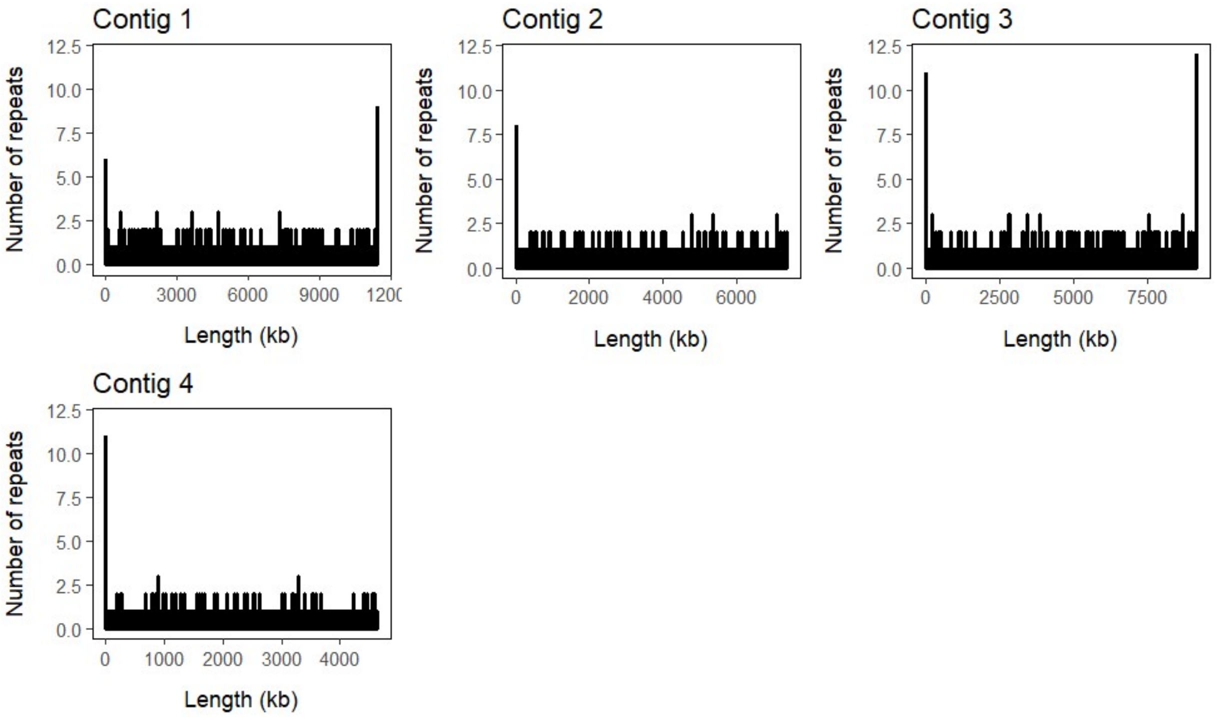

Figure S15: Overview of telomeric regions of a contig in slidning window of 100 b with 25 b increments. These contigs are from the draft made for Penicillium aurantiogriseum with 75x coverage. Contigs comprising mtDNA or exclusively rRNA genes are not included in the analysis.

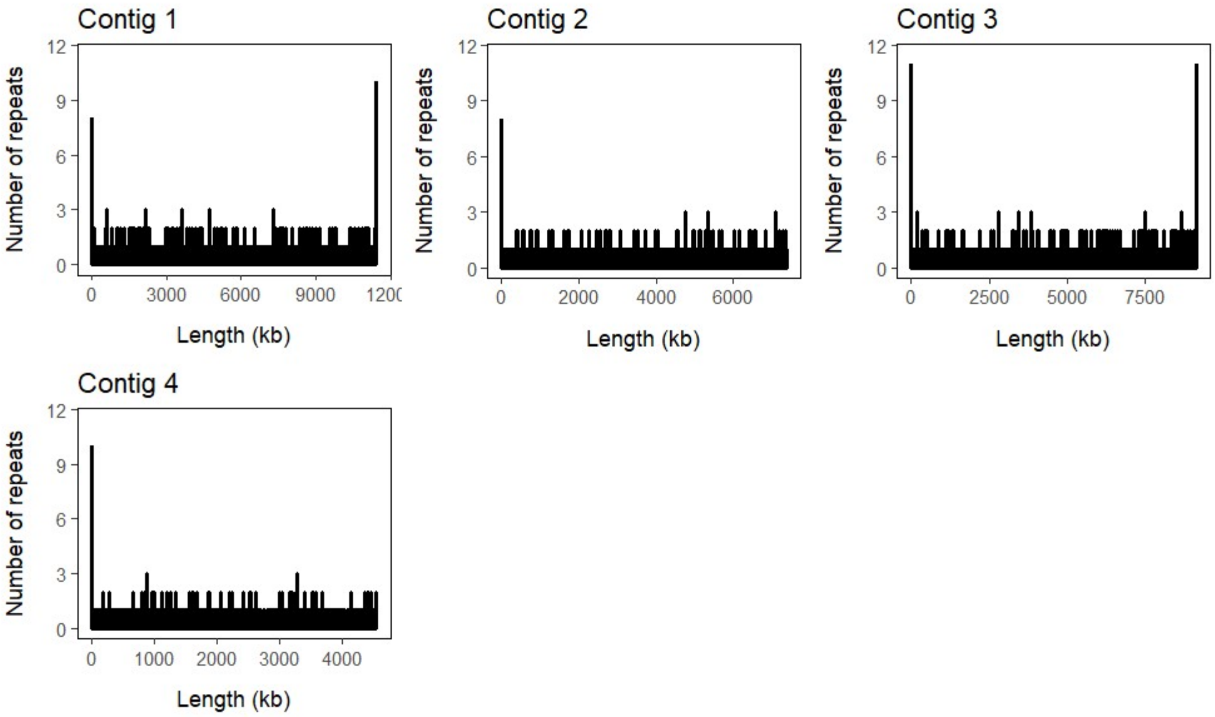

Figure S16: Overview of telomeric regions of a contig in slidning window of 100 b with 25 b increments. These contigs are from the draft made for Penicillium aurantiogriseum with 50x coverage. Contigs comprising mtDNA or exclusively rRNA genes are not included in the analysis.

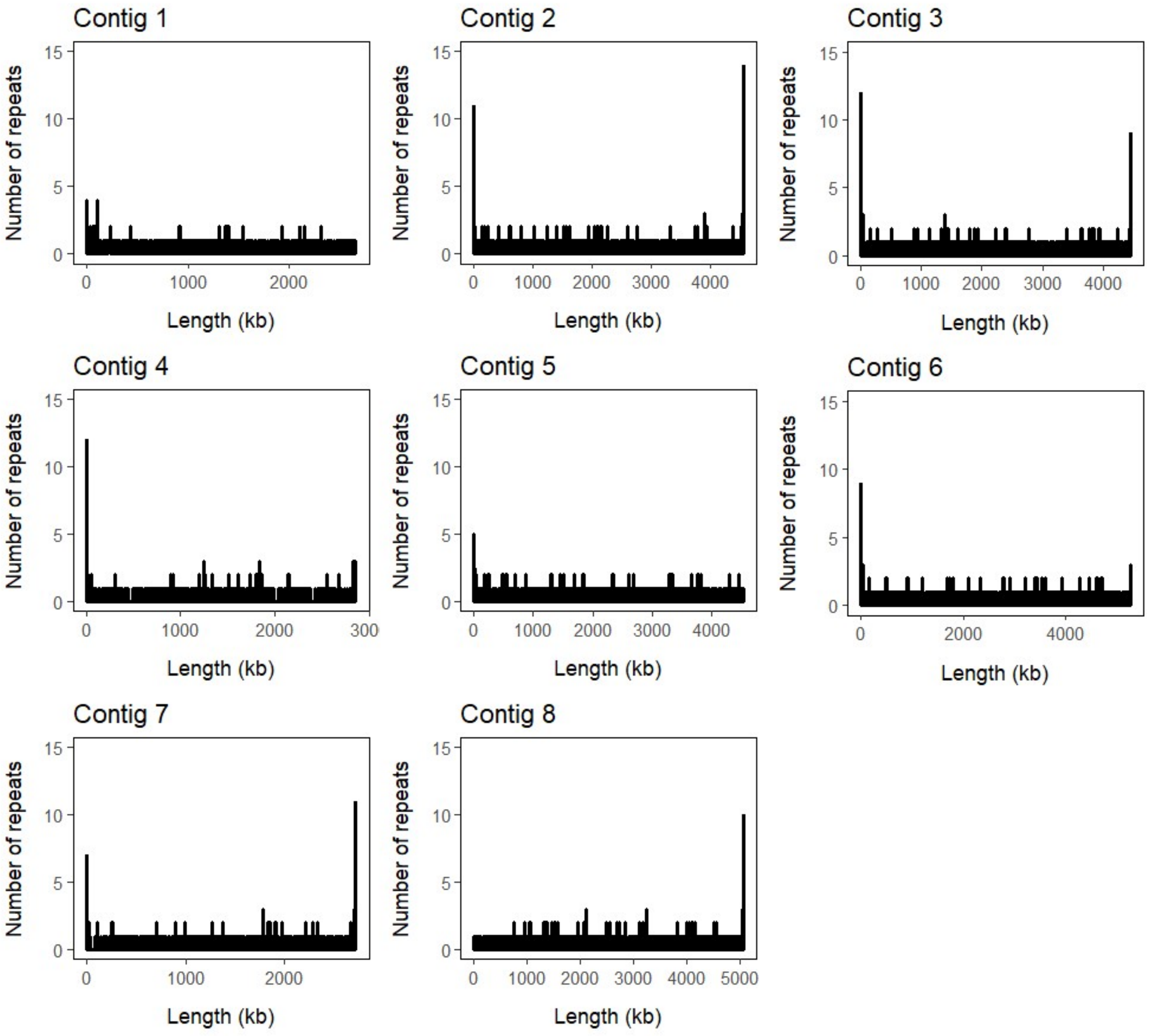

Figure S17: Overview of telomeric regions of a contig in slidning window of 100 b with 25 b increments. These contigs are from the draft made for Aspergillus sp. (subgen. Cremei) with 91x coverage. The increase of number of repeats in the start of contig 5 is not continuous and is therefore not interpreted as a telomeric region. Contigs comprising mtDNA or exclusively rRNA genes are not included in the analysis.

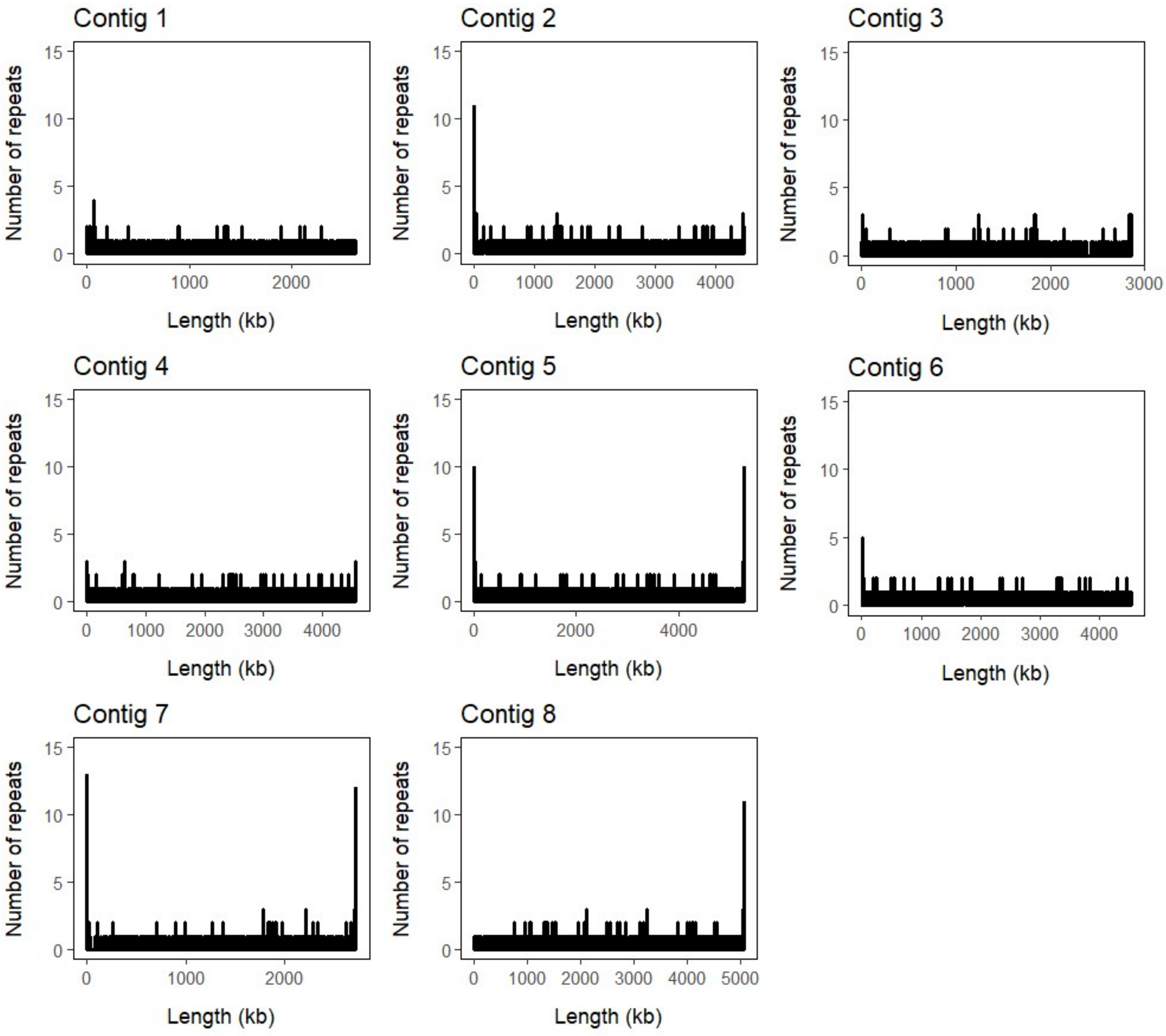

Figure S18: Overview of telomeric regions of a contig in slidning window of 100 b with 25 b increments. These contigs are from the draft made for Aspergillus sp. (subgen. Cremei) with 75x coverage. The increase of number of repeats in the start of contig 6 is not continuous and is therefore not interpreted as a telomeric region. Contigs comprising mtDNA or exclusively rRNA genes are not included in the analysis.

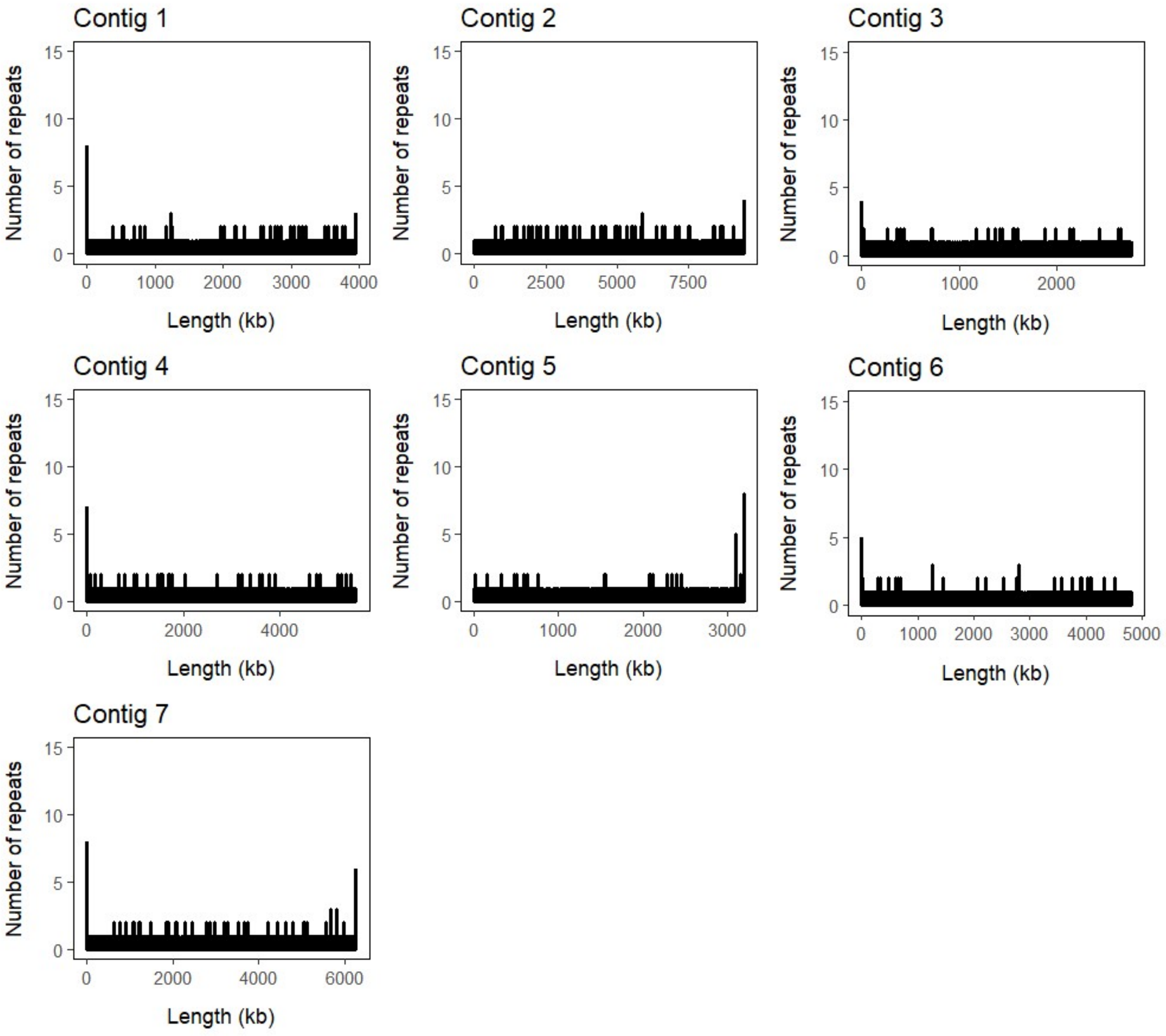

Figure S19: Overview of telomeric regions of a contig in slidning window of 100 b with 25 b increments. These contigs are from the draft made for Aspergillus sp. (subgen. Cremei) with 50x coverage. Contigs comprising mtDNA or exclusively rRNA genes are not included in the analysis.
